# Transcriptomic Profiling of Psoriatic Lesions by Tape-Stripping Reveals Site-Specific Differences

**DOI:** 10.3390/jcm15114034

**Published:** 2026-05-22

**Authors:** Maruška Marovt, Martina Krušič, Mario Gorenjak, Pij Bogomir Marko, Uroš Potočnik

**Affiliations:** 1 Department of Dermatovenerology, University Medical Centre Maribor, 2000 Maribor, Slovenia; maruska.marovt@gmail.com (M.M.);; 2 Faculty of Medicine, University of Maribor, 2000 Maribor, Slovenia; 3 Faculty of Chemistry and Chemical Engineering, University of Maribor, 2000 Maribor, Slovenia; 4 Department for Science and Research, University Medical Centre Maribor, 2000 Maribor, Slovenia

**Keywords:** psoriasis, tape-stripping, differential gene expression, skin transcriptome

## Abstract

**Background/Objectives**: Psoriasis is a chronic immune-mediated skin disease, with plaque psoriasis being its most prevalent form. Although biologic therapies have significantly improved outcomes for patients with moderate to severe disease, certain anatomical regions often remain resistant to treatment. Given the observed variability in treatment response depending on lesion location, we aimed to explore anatomical site-specific differences in the skin transcriptome. **Methods**: Using a non-invasive tape-stripping technique, we collected and analyzed 44 psoriatic plaque samples from the scalp, trunk, upper extremities, and lower extremities, followed by differential gene expression and gene ontology analysis. Moreover, we included 80 samples obtained from healthy skin biopsies (GSE54456) to conduct an inflammation-controlled approach. We used two different approaches, an intra-disease approach, in which anatomically different sites were compared, and an inflammation-controlled approach, in which the inflammation bias was reduced. **Results**: Our findings indicate distinct molecular signatures and biological pathways across different anatomical sites, including differential expression of SERPINB7 and miRNAs such as miR-205 and miR-203a, along with different pathways. Furthermore, our results emphasized the heterogeneity of psoriasis and suggested that site-specific molecular mechanisms may contribute to variations in disease manifestation and treatment response. **Conclusions**: This study highlights the need for more personalized, site-specific therapeutic strategies. It should be considered an exploratory pilot study, and larger studies with paired samples from multiple anatomical sites within the same patients are needed to validate the identified transcriptomic signatures.

## 1. Introduction

Psoriasis is a chronic, immune-mediated skin disease that affects approximately 125 million people worldwide [1,2]. More than 80% of psoriasis cases are plaque psoriasis, characterized by erythematous, scaly patches and plaques commonly on extensor surfaces. Plaque psoriasis is associated with several comorbidities including psoriatic arthritis, cardiometabolic diseases, and depression [3]. People with moderate to severe psoriasis are usually treated with biologics, which are considered very effective [4]. However, there are known areas that are difficult to treat, such as the scalp, nails, palms, and soles [5,6]. In addition, psoriasis on the legs has been shown to be resistant to treatment in some cases and has recently been recognized as a new difficult-to-treat area [7,8,9,10]. Moreover, studies have also shown that the response to treatment varies between different anatomical sites [3,11].

The understanding of the pathogenesis of psoriasis is mainly based on studies performed on skin biopsies, which provide a deep insight into the cellular and molecular mechanisms of inflammation. In recent years, the use of non-invasive sampling of skin lesions with adhesive patches has increased substantially [12,13,14,15,16], as this approach enables simple collection of tissue material for various analyses and provides insight into key molecular processes in the skin. Over the last decade, techniques such as tape-stripping have increasingly been used for the assessment and characterization of proteins [12], analysis of molecular changes in the skin [13], and investigation of the transcriptomic profile of psoriatic skin [14]. Due to the minimally invasive nature of skin tissue collection [17], this method has become an important research tool for studying various skin diseases, including psoriasis [13,14] and AD [18,19], although sampling is mainly limited to removal of the stratum corneum [15,20]. Results from transcriptomic and proteomic analyses obtained using adhesive patches confirm the usefulness of the technique for detecting key immune and barrier-related biomarkers; however, several studies have also highlighted important limitations associated with tape-based skin sampling, including lower yield and greater variability of the obtained data. A particular challenge is the low quantity of RNA, which is often highly degraded, thereby complicating downstream analyses [19,21,22].

Various transcriptomic studies have been conducted comparing anatomically different sites of inflamed skin [7,23,24,25], but there is still a lack of evidence comparing the transcriptomic profiles of plaques at different anatomical locations. Therefore, we aimed to perform a body site-specific transcriptomic study using the non-invasive tape-stripping technique to analyze differences in gene expression signatures across four distinct affected sites in psoriasis: the scalp, trunk, lower, and upper extremities.

## 2. Materials and Methods

### 2.1. Study Population and Characteristics

In total, twenty-four adult patients with chronic plaque psoriasis were enrolled in the present study. All patients were recruited at the Department of Dermatovenerology, University Medical Centre Maribor, Slovenia. The present study was evaluated and approved on 6 May 2021 by the Medical Ethics Committee of the University Medical Centre Maribor (approval number UKC-MB-KME-37/21). The study was conducted in accordance with the Declaration of Helsinki. All participants received verbal information from the attending physician and provided written informed consent prior to enrolment in the study. All enrolled patients were of Caucasian Central European ethnicity. Only individuals with chronic plaque psoriasis lasting at least six months who were naïve in respect to biological treatment were included in the present study. The exclusion criteria included immunodeficiency; the use of systemic treatment; and the use of any topical anti-inflammatory agents within the four weeks prior to enrollment. A summary of patient characteristics is provided in Supplementary Table S1.

### 2.2. Sample Collection and RNA Extraction

From a representative sample of lesional skin, D-Squame tape strips (CuDerm, Dallas, TX, USA) were used to collect skin samples from one to four body sites, depending on the patient’s clinical presentation. These body sites included the trunk (T), scalp (S), upper (UL), and lower extremities (LL). The clinical presentation among patients was highly heterogeneous, which prevented consistent sample collection from all four anatomical sites in each patient. In total, 44 psoriatic skin samples were collected. Specifically, eighteen samples were obtained from the trunk, six from the scalp, eleven from the upper extremities, and nine from the lower extremities. Full information on patients’ samples per body site with details on sample quality and quantity are provided in Supplementary Table S2.

The skin was first wiped clean with an alcohol swab, and the first tape strip was discarded. Subsequently, ten consecutive tape strips were collected from representative lesions by applying pressure with the fingers to the same skin area. Immediately after collection, the tape strips were placed in a stabilization buffer (1 mL DNA/RNA Shield, Zymo Research) until RNA extraction. RNA was extracted using an miRNeasy Mini Kit (Qiagen, Hilden, Germany) and stored at −80 °C until further processing.

### 2.3. RNA Sequencing of Psoriatic Samples

For psoriatic samples, both lncRNA and mRNA 100 bp paired-end libraries were constructed using the Hieff NGS™ MaxUp Human rRNA Depletion Kit (rRNA & ITS/ETS), paired with the Hieff NGS™ Ultima Dual-mode mRNA Library Prep Kit at BGI facilities (BGI, Hong Kong, China). Raw data with adapter sequences or low-quality sequences were filtered using SOAPnuke software by BGI [26].

### 2.4. GEO Database

Raw data from 80 healthy tissue samples were acquired from the Gene Expression Omnibus (GEO) database, specifically from dataset GSE54456. The selected samples are provided in Supplementary Table S3. Detailed descriptions of the sampling procedures, RNA library preparation, and sequencing methods utilized for this dataset are provided in an article by Li and colleagues [27].

### 2.5. RNA-seq Analysis

In total, 44 psoriatic samples and 80 samples from healthy controls (HCs) were included in the RNA-seq analysis using two approaches. In the intra-disease approach, differential gene expression analysis was performed to compare plaques from different anatomical sites. In the inflammation-controlled approach, differential gene expression analysis was first performed between psoriatic lesions and the skin of healthy controls to identify differentially expressed genes (DEGs) associated with psoriasis-related inflammation. These DEGs were subsequently excluded from the raw count matrix of psoriatic samples prior to downstream analyses in order to reduce the dominant inflammation-associated transcriptional signal and enrich for residual anatomical site-specific expression patterns. Specifically, genes meeting the significance criteria of an unadjusted *p*-value < 0.05 and log_2_ fold change (log_2_FC) ≥ 2 or ≤−2 were removed. Subsequently, site-specific gene expression analyses—including comparisons between the trunk, scalp, upper extremities, and lower extremities—were performed on the filtered gene subset, enabling the identification of site-specific gene expression signatures with reduced inflammation bias.

RNA-seq analysis was carried out for each approach separately to ensure analysis batch normalization. Data analysis was conducted using the R 4. 4. 2 environment (R Core Team 2024, Vienna, Austria). Raw reads from all samples were aligned to the hg19 reference genome using the *Rsubread 2. 18. 0* R package [28]. The mapped reads were then quantified and assigned to genomic features using *featureCounts* [29]. Counts per million (CPMs) were calculated using the *edgeR 4. 2. 2* R package [30]. Genes with low expression were excluded using CPM thresholds corresponding to a read count of 2, which was arbitrarily set due to the low-yield nature of the tape-stripping technique. Retained genes were normalized using the trimmed mean of M values method (TMM) [31]. Subsequently, mean–variance modeling at the observational level transformation (VOOM) was applied [32]. Differential expression was determined using linear models adjusted for batch effect and empirical bayes implemented in the *limma 3. 60. 6* R package [33]. DEGs were considered significant at an unadjusted *p*-value < 0.05 and log_2_FC ≥1 or ≤−1.

The results of the RNA-seq analysis were visualized using a volcano plot and a heatmap. In the volcano plot, genes with a *p*-value < 0.05 and a log_2_FC ≥ 1 or ≤−1 were highlighted. For the heatmap analysis, the top 50 DEGs were selected, and z-scores were calculated to enable comparison of relative gene expression patterns across samples.

### 2.6. Gene Ontology Analysis

Gene ontology (GO) analysis was conducted separately for each approach. For the identified differentially expressed genes, GO analysis was performed using the Enrichr database [34,35,36] to identify enriched GO terms related to molecular functions and biological processes. The input genes were selected to include the top 100 most relevant genes.

## 3. Results

### 3.1. Intra-Disease Approach

Differential expression analysis revealed 1200 DEGs in T versus UL (259 down- and 941 upregulated genes), 2088 DEGs in T versus LL (819 down- and 1269 upregulated genes), 1013 DEGs in T versus S (828 down- and 185 upregulated genes), 486 DEGs in LL versus UL (87 down- and 399 upregulated genes), 1626 DEGs in S versus UL (380 down- and 1246 upregulated genes), and 1483 DEGs in S versus LL (265 down- and 1218 upregulated genes). All DEGs are provided in Supplementary Table S4. Volcano plots and heatmaps are shown in Supplementary Figures S1–S12. Using the results obtained from RNA-seq analysis, GO analysis was performed for biological processes and molecular functions (Supplementary Table S5). Significant enriched GO terms and associated genes for each pairwise comparison are presented in Supplementary Table S5.

GO analysis of psoriatic skin lesions identified enrichment in terms related to epidermal and epithelial development, including epidermis development, keratinocyte differentiation, and epithelium development. Functional categories such as serine-type endopeptidase inhibitor activity, phospholipase activity, and lipid transport were also significantly represented. Additional enrichment was observed in signaling and ion transport functions, including G protein-coupled receptor binding and potassium channel activity.

### 3.2. Inflammation-Controlled Approach

Firstly, RNA-seq analysis between psoriatic lesions and HCs was conducted to reduce potential inflammation bias. All DEGs are provided in Supplementary Table S6. Volcano plots and heatmaps are provided in Supplementary Figures S13–S20. Subsequently, significant DEGs were excluded from the raw counts of psoriatic samples, and site-specific differential gene expression analysis was performed. The excluded unique DEGs are provided in Supplementary Table S7.

Differential expression analysis revealed 407 DEGs in T versus UL (89 down- and 318 upregulated genes), 732 DEGs in T versus LL (373 down- and 359 upregulated genes), 295 DEGs in T versus S (225 down- and 70 upregulated genes), 219 DEGs in LL versus UL (32 down- and 187 upregulated genes), 589 DEGs in S versus UL (134 down- and 455 upregulated genes), and 563 DEGs in S versus LL (156 down- and 407 upregulated genes). All DEGs are provided in Supplementary Table S8. Volcano plots and heatmaps are provided in Supplementary Figures S21–S32. Using the results obtained from RNA-seq analysis, we performed GO analysis for biological processes and molecular functions (Supplementary Table S9). Significant enriched terms and genes associated with each GO term for each individual comparison are presented in Supplementary Table S9.

GO analysis of psoriatic skin lesions revealed differential enrichment of biological processes related to tissue development, immune response, and signal transduction across anatomical sites. Core functional categories included lipid transport, receptor binding, ion channel activity, and enzyme regulation. Terms associated with cellular communication, transcriptional regulation, and metabolic processes were also significantly represented.

## 4. Discussion

To the best of our knowledge, this is the first study to compare anatomical site-specific psoriatic lesions using two different approaches. By using the intra-disease approach, we explored the variability and unique gene expression patterns among individual site-specific psoriatic lesions, while the inflammation-controlled approach provided a normalized baseline in which inflammation-related genes were diminished by filtering DEGs obtained from comparisons between psoriatic lesions and healthy tissues, and then applying the analysis excluding these DEGs to the psoriatic lesions. In that way, we ensured that the comparisons were grounded in the disease-specific context, while reducing potential inflammation-related bias. However, as some genes may be associated with both inflammatory processes and anatomical specificity, this approach should be interpreted as a strategy for partial control of inflammatory bias rather than its complete elimination.

Initially, a comparison between the trunk and upper extremities was performed. Small proline-rich protein (SPRR) genes were found to be involved in the top biological processes identified in the intra-disease approach. These processes are primarily related to skin development and keratinocyte differentiation. Genes from this family were also significantly upregulated in our RNA-seq analysis (Supplementary Table S4). It has been shown that the expression of these genes is linked to keratinocyte terminal differentiation both in vivo and in vitro [37]. In the molecular function GO analysis, the term “serine-type endopeptidase inhibitor activity” was observed, which includes a family of serine protease inhibitors (SERPINs). Studies have shown that SERPINB4 is upregulated in psoriatic skin and serum [38,39,40]. Interestingly, a study by Zhang and colleagues [41] suggests that SERPINB4 has a role in the promotion of keratinocyte inflammation via activation of p38MAPK. Recently, researchers have also demonstrated higher expression of SERPINB3/B4 in the lesions of psoriatic individuals [42]. Moreover, they suggest that SERPINB3/B4 may stimulate nuclear factor kappa (NF-κB) signaling [42]. Furthermore, a study by Ayvaz and colleagues observed that SERPINB7 was significantly downregulated in psoriatic skin compared to healthy controls [43]. Conversely, another study has shown that the expression levels of *SERPINB7* were increased in psoriatic lesions in comparison with healthy tissue and non-lesional skin [44]. Concomitantly, our results also showed that *SERPINB7* is upregulated in certain anatomical sites of psoriatic lesions, particularly the trunk compared to the lesions on the upper and lower extremities, and the scalp compared to the lower extremities. Similarly, our findings showed increased expression of *SERPINB7* in trunk and scalp lesions compared to healthy skin. These findings suggest that higher expression of *SERPINB7* is not uniform across all psoriatic lesions and may be involved in anatomical site-specific mechanisms, especially in trunk and scalp lesions.

On the other hand, the inflammation-controlled approach, which compared the trunk and upper extremities, highlighted a term associated with Hippo signaling, which was also observed in the comparison between the scalp and the lower extremities. Evidence has shown the implication of dysregulation of the Hippo-yes-associated protein (Hippo-YAP) pathway in psoriasis [45]. A recent study showed that YAP1 plays a key role in psoriasis by enhancing keratinocyte proliferation and inflammation, and its expression is significantly increased by psoriasis-related inflammatory signals [46]. Notably, the inhibition of YAP1 function has been shown to alleviate psoriatic symptoms, suggesting its potential as a therapeutic target, although further studies are needed to confirm its clinical relevance [46]. In addition, MST1, an upstream kinase of the Hippo pathway, and TEAD4, an important downstream transcription factor of the Hippo pathway, were found to be upregulated in psoriatic lesions compared to healthy skin [47,48]. Interestingly, TEAD4 has been shown to transcriptionally regulate the expression of SERPINB3/4 [48], which may point to a potential link between Hippo signaling and the differential expression of SERPINB family members observed in our study.

Next, a comparison between the trunk and lower extremities was performed. The terms “keratinocyte differentiation” and “epidermis development” were identified as key results of the intra-disease approach, emphasizing their role in psoriatic skin. These terms are commonly enriched in psoriatic skin [49,50]. Moreover, the inflammation-controlled approach highlighted the term “acetylcholine binding”. A study by Chen and colleagues [51] demonstrated increased expression of the α7 subunit of the nicotinic acetylcholine receptor (α7nAChR) in keratinocytes of psoriatic lesions compared to healthy skin. More intriguingly, their results also suggest that activation of this receptor can reduce psoriatic lesions [51]. To further explore the complexity of psoriasis and its varied clinical presentation, a separate study investigated differences between psoriatic plaques on the trunk and lower extremities, identifying thirteen epidermal and dermal differences between these anatomical sites [7]. Notably, they emphasized three statistically significant immunohistochemical markers that distinguish lesions on the trunk and lower extremities [7]. They demonstrated that the expression ratio of cytokeratin (CK) 10 and the antimicrobial peptide psoriasin (S100A7) was increased in biopsies taken from the lower leg compared with trunk lesions, whereas the expression of Bcl-2alpha was reduced in trunk lesions compared to those on the lower extremities [7].

Additionally, a comparison between the trunk and the scalp was performed. In the intra-disease approach, genes from the keratin (*KRT*-) family were mostly involved in biological processes related to cellular structural organization (e.g., “intermediate filament organization” and “supramolecular fiber organization”) and epithelial processes (e.g., “epithelial cell differentiation” and “epithelium development”). A study published by Ahn and colleagues examined gene expression profiles in conventional, scalp, and palmoplantar psoriasis [24]. Their research found that KRT and keratin-associated protein (KRTAP) family genes were highly expressed in scalp psoriasis and, in many cases, differentially expressed compared to control skin. These genes are likely important in the normal development of hair follicles and hair [24]. Our findings align with those of the previously published article. Moreover, the comparison also identified the GO molecular function term “frizzled binding”, involving *WNT* genes. Several studies have demonstrated that Wnt signaling is altered in psoriatic skin compared to normal skin [52,53], and that expression levels of Wnt5a are increased in psoriatic skin [53]. However, further research is needed to elucidate any anatomical differences in Wnt5a expression across various psoriatic lesions.

A comparison between the lower and upper extremities was also performed. Interestingly, the GO results from both the intra-disease and inflammation-controlled approaches indicated the involvement of voltage-gated sodium channels in psoriatic lesions. It is known that ion channels play a vital role in maintaining the homeostasis of the skin. Recently, it has been reported that the expression of voltage-gated sodium ion channel Nav1.8 is increased in the epidermis of inflammatory skin diseases such as rosacea and psoriasis, both in patients and mouse models [54]. The study revealed that silencing Nav1.8 reduced disease symptoms, inflammation, the expression of pro-inflammatory cytokines (IL1β, IL6), and the accumulation of reactive oxygen species (ROS), indicating a key role for Nav1.8 in regulating inflammation in keratinocytes [54]. They also demonstrated an intriguing connection between tumor necrosis factor α (TNFα), a cytokine that plays a key role in driving skin inflammation associated with psoriasis, and Nav1.8. Their results indicated that TNFα stimulated an increase in Nav1.8 expression in keratinocytes [54]. In line with these observations, our results suggest the potential involvement of sodium channels in lesions located on the extremities. However, it remains unclear whether ion channel expression differs significantly across anatomical locations. Importantly, these computational findings, including the inferred involvement of ion channels, have not been independently experimentally validated and should therefore be considered hypothesis-generating rather than definitive evidence.

When a comparison between lesions on the scalp and lower extremities was conducted, the intra-disease approach showed involvement of sphingolipid and ceramide metabolic processes. Lipids play an important role in the skin, especially when they are transformed into bioactive mediators [55]. Bioactive mediators include sphingolipids [56], which influence keratinocyte growth [57]. Previous studies have shown that levels of bioactive lipids in psoriatic lesions are altered [58,59]. Interestingly, a recent study investigated the lipid skin profile of psoriatic patients treated with guselkumab using the tape-stripping method [60]. Their results showed that treatment with guselkumab normalized the ceramide (a type of sphingolipid) profile [60]. Likewise, our comparison between the scalp and the upper extremities using the inflammation-controlled approach identified the GO molecular function of ceramide 1-phosphate (C1P) transfer activity. A study by Panpan and colleagues [58] showed that most ceramides were upregulated in psoriatic lesions compared to non-lesional skin, and they highlighted a potential role of C1P as a novel therapy target for psoriasis. Their study showed that C1P exacerbates psoriasis-like inflammation, and its suppression could help to alleviate inflammation [58].

Moreover, our inflammation-controlled approach, which compared the scalp and the lower extremities, revealed ephrin receptor activity. The Eph/Ephrin system is activated during various skin pathologies, including psoriasis [61]. Evidence showed that *EPHA2* expression is increased in psoriatic plaques compared to healthy skin [61]. In contrast, mRNA transcripts for epidermal ephrin-A ligands (*EFNA1*, *EFNA3*, *EFNA4*) were decreased in psoriatic plaques [61]; however, there is no evidence of differences in ephrin receptors between different anatomical lesions.

Of note, in 2018, the first study that conducted a transcriptomic comparison of intra- and inter-individual variability in inflamed skin across anatomically different sites was published [25]. The authors recruited five psoriatic patients who had plaques on different anatomical sites, including the arm, abdomen, leg, thigh, back, and buttock, depending on each patient’s clinical characteristics. The results showed that the transcriptomes of inflamed skin from the same individual tended to group together, regardless of the anatomical location [25]. Furthermore, another study by Tsoi and colleagues [13] explored the transcriptome of psoriasis obtained from tape strips. The findings showed that the tape-stripping technique is particularly effective for sampling non-lesional skin, as it allows for the detection of a pre-psoriatic state that cannot be identified by full-thickness skin biopsies. Moreover, their results demonstrate that the transcriptome obtained from tape strips is a reliable method for obtaining the transcriptome of the upper layers of the epidermis [13]. They also reported that some DEGs are more dysregulated in tape-stripped lesional skin samples compared to full-thickness biopsy samples, for instance the genes *S100A12*, *DEFB4*, *SPRR2B*, and *SPRR2A* [13].

Notably, most studies had primarily looked at differences between lesional skin and non-lesional or healthy skin, with most studies obtaining the results from skin biopsies [23,24]. The study by Ahn and colleagues [24] reported an RNA-seq analysis on eight patients with conventional plaque psoriasis on the trunk and extremities, eight patients with scalp psoriasis, and three patients with palmoplantar psoriasis. The authors compared skin biopsies from different anatomical sites with normal healthy skin samples. The top upregulated genes in conventional plaque psoriasis included *SST*, *TTTY14*, *PRKY*, *IL20*, *KRT33A*, and *HOXD11*, while the top downregulated genes included *SERTM1*, *ADAMTS16*, *MATN4*, and *HAS1* [24]. Our RNA-seq results indicated that *IL20* is upregulated in all four comparisons (T versus HC, UL versus HC, LL versus HC, and S versus HC). In contrast, *KRT33A* was found to be downregulated in the trunk compared to HCs, but upregulated in lower extremities and scalp lesions compared to HCs. The highlighted downregulated genes in the study by Ahn were also downregulated in our study, where the trunk and extremity lesions were compared with HCs. In contrast, the HAS1 gene is upregulated in all our comparisons. Upon examination of the scalp transcriptome, the authors identified the top upregulated genes *LCE3C*, *FAM26D*, and *CBLN2*, and the top downregulated genes *AGR3*, *TBX5*, and *HOXA10*. Our results showed that *LCE3C* is upregulated in the trunk, the lower extremities, and scalp lesions compared to HCs, but not in the upper extremities. Interestingly, our results are contradictory, since we did not observe the genes *AGR3*, *TBX5*, and *HOXA10* in scalp lesions compared to HCs. These genes were found to be downregulated in the trunk, upper, and lower extremities in comparison to HCs. Moreover, the gene *CBLN2* was downregulated in our RNA-seq analysis in the trunk, upper and lower extremities in comparison to HCs. Furthermore, a study by Ruano and colleagues [23] evaluated similarities and dissimilarities between the scalp and the skin transcriptome. The transcriptome of scalp psoriasis showed stronger changes in the expression of several gene sets, especially those triggered by interferon-gamma, compared to skin psoriasis. In contrast, skin psoriasis was primarily linked to the activation of genes involved in the keratinocyte response to TNFα, IL-17, and IL-22 [23]. They reported that the gene *CXCL9* is represented in skin psoriasis and is highly upregulated in scalp lesions. Our study indicated contradictory results, with the gene *CXCL9* downregulated in the scalp compared to the upper extremities (Supplementary Table S4). This discrepancy may reflect both biological heterogeneity and methodological differences between studies. Notably, the referenced study used full-thickness punch biopsies collected from infiltrated plaque borders, while our study employed tape stripping, which selectively samples superficial epidermal layers. Consequently, differences in tissue depth, cellular composition, and local inflammatory infiltrates may substantially affect the detected transcriptomic signatures. Further studies directly comparing sampling methodologies will be necessary to clarify the role of *CXCL9* across anatomical sites in psoriasis.

Our RNA-seq analysis also suggests differentially expressed micro RNAs (miRNAs). MiRNAs are small RNAs involved in various biological processes, including development, differentiation, and disease progression, by modulating the expression of target genes [62]. They also play an important role in multiple skin diseases, including psoriasis [63,64]. Importantly, for many of the expressed miRNAs in the present study, no association with psoriasis has been described so far.

Interestingly, we discovered that the levels of miR-205 were downregulated in the trunk compared to the scalp and upregulated in the scalp compared to the upper extremities, in both intra-disease and inflammation-controlled approaches. However, in other comparisons, this miRNA was not significantly expressed. A recent study by Mihu and colleagues [65] showed increased levels of miR-205 in peripheral blood samples of psoriatic patients already treated with anti-TNFα, compared to those who had not received therapy. They suggested a potential link between the increased expression of miR-205 and the decreased levels of TNF-α in treated psoriasis patients. On the other hand, another study [66] found reduced levels of miR-205 in psoriatic skin compared to healthy skin. Moreover, their results suggested a protective role of miR-205 through deactivation of the Wnt/β-catenin and MAPK signaling pathways. Additionally, the findings from Zibert and colleagues [67] indicate the possible involvement of miR-205 in the regulation of the Akt signaling pathway, potentially contributing to reduced apoptosis in psoriasis. Taken together, the role of miR-205 in psoriasis remains controversial and unclear; therefore, further studies are needed to clarify its importance in this disease.

Moreover, our intra-disease approach indicated upregulation of miR-203a in the trunk compared to the upper and lower extremities (Supplementary Table S4). A study investigating miRNA profiles in skin and serum samples from atopic dermatitis and psoriatic patients validated several miRNAs, including miR-203a [68]. Interestingly, punch skin biopsy results from psoriatic lesions on the upper extremities showed increased expression of miR-203a compared with healthy skin [68].

## 5. Conclusions

To conclude, by integrating the findings from both the intra-disease and the inflammation-controlled approach, we obtained a more comprehensive understanding of both the shared and the site-associated aspects of psoriatic lesions. Nevertheless, several limitations of the present study should be acknowledged. These include the relatively small overall sample size, the limited and imbalanced number of samples across specific anatomical sites, and the absence of paired samples collected from multiple anatomical locations within the same individuals. Consequently, substantial inter-individual variability may have influenced the observed transcriptomic differences, thereby limiting the statistical power to confidently attribute these findings exclusively to anatomical site-specific effects. Therefore, the identified site-associated transcriptional patterns should be interpreted cautiously and considered hypothesis-generating rather than definitive evidence of true anatomical specificity. Importantly, the present work should be regarded as an exploratory pilot study, and larger studies including paired samples from multiple anatomical sites within the same patients will be necessary to validate the identified transcriptomic signatures. In addition, the tape-stripping technique yields relatively low amounts of RNA, necessitating less stringent gene-filtering thresholds, which may further affect transcriptomic sensitivity and variability.

Additionally, the reliance on an external cohort represents an important limitation of the present study. Despite applied normalization, potential clinical and technical biases related to differences in sampling procedures, RNA extraction methods, sequencing protocols, and cohort characteristics cannot be completely excluded. Therefore, part of the observed transcriptomic variation may reflect inter-cohort methodological differences rather than exclusively anatomical site-specific biological effects.

To the best of our knowledge, our study is the first to compare four different anatomical psoriatic lesions using the non-invasive tape-stripping technique. Our results highlight the differences between various anatomical sites and suggest the involvement of miRNAs. Further investigation of the localized expression patterns between different anatomical lesions could provide insights into the mechanisms underlying site-specific manifestations of psoriasis and potentially lead to site-specific treatment. While some of the GO terms and miRNAs have known associations with psoriasis, their specific roles in specific anatomical sites are not extensively documented. Therefore, further research is needed to understand how different psoriatic anatomical sites differ from each other. Additionally, we suggest that site-specific diagnostics could help in achieving better treatment response, specifically for difficult-to-treat psoriatic lesions.

## Data Availability

The datasets generated and analyzed during the current study are available in the Gene Expression Omnibus (GEO) repository, GSE312703.

## Supplementary Materials

The following supporting information can be downloaded at https://www.mdpi.com/article/10.3390/jcm15114034/s1: Table S1: Characteristics of psoriatic patients; Table S2: Quantity and quality of RNA isolated from tape strips; Table S3: Selected samples of healthy biopsies (RUN names); Table S4: Differentially expressed genes from the intra-disease approach; Figures S1–S12: Volcano plots and heatmaps for differentially expressed genes from the intra-disease approach; Table S5: Gene ontology enrichment analysis for the intra-disease approach; Table S6: Differentially expressed genes in comparisons between psoriatic lesions and healthy controls; Figures S13–S20: Volcano plots and heatmaps for differentially expressed genes between psoriatic lesions and healthy controls; Table S7: Excluded unique differentially expressed genes; Table S8: Differentially expressed genes from the inflammation-controlled approach; Figures S21–S32: Volcano plots and heatmaps for differentially expressed genes from the inflammation-controlled approach; Table S9: Gene ontology enrichment analysis for the inflammation-controlled approach.

## Abbreviations

The following abbreviations are used in this manuscript:

LL	lower extremity
UL	upper extremity
S	scalp
T	trunk
