# Supplementary material for: Transcriptomic Profiling of Psoriatic Lesions by Tape-Stripping Reveals Site-Specific Differences
Source: J Clin Med. 2026 May 22;15(11):4034. doi: 10.3390/jcm15114034 (PMC13258650; doi:10.3390/jcm15114034)
Supplement: Supplementary file 1 [file jcm-15-04034-s001.zip › Supplementary Figures S1 - S12.pdf]

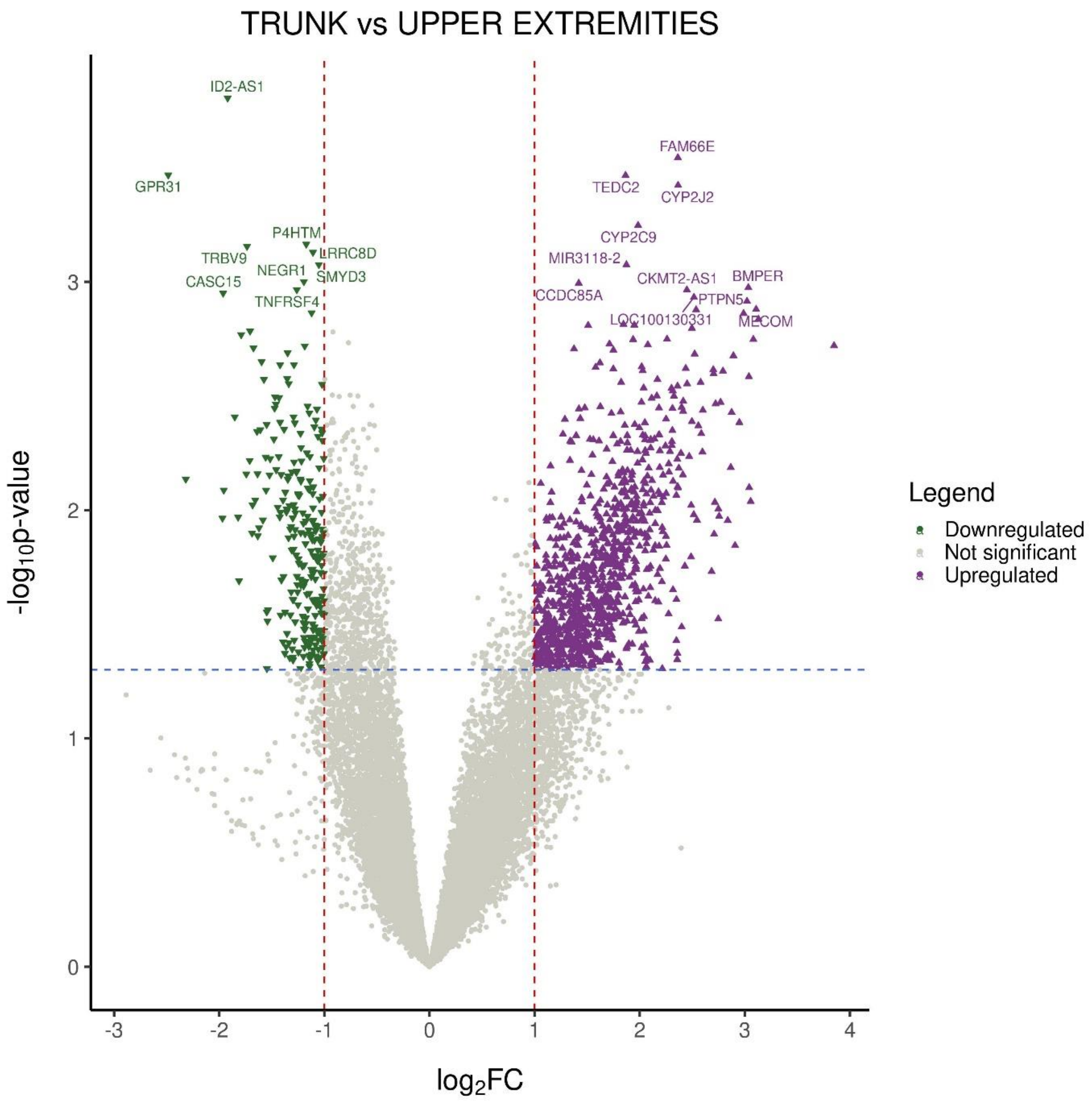

Figure S1: Volcano plot for T vs UL comparison.

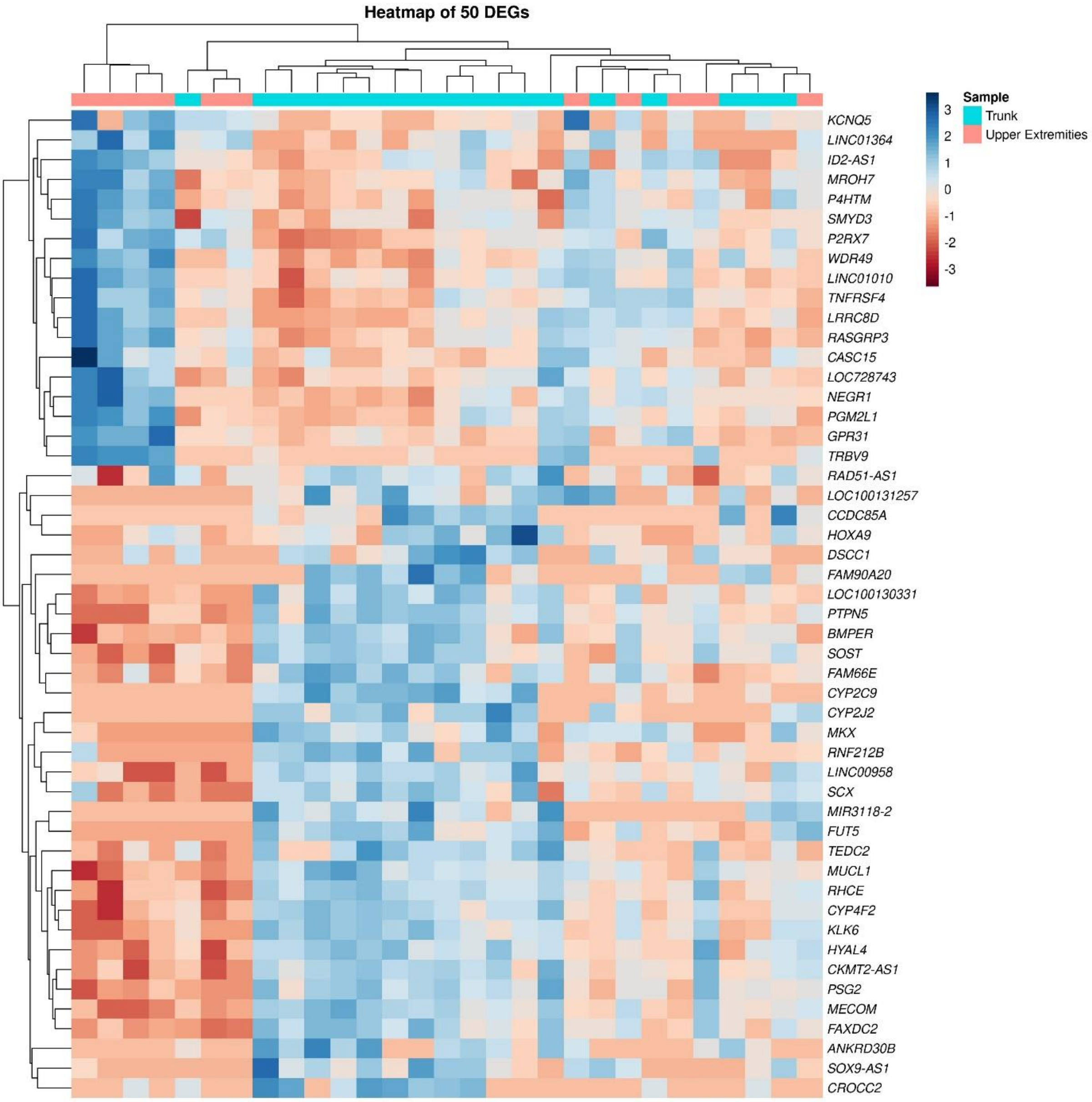

Figure S2: Heatmap for T vs UL comparison.

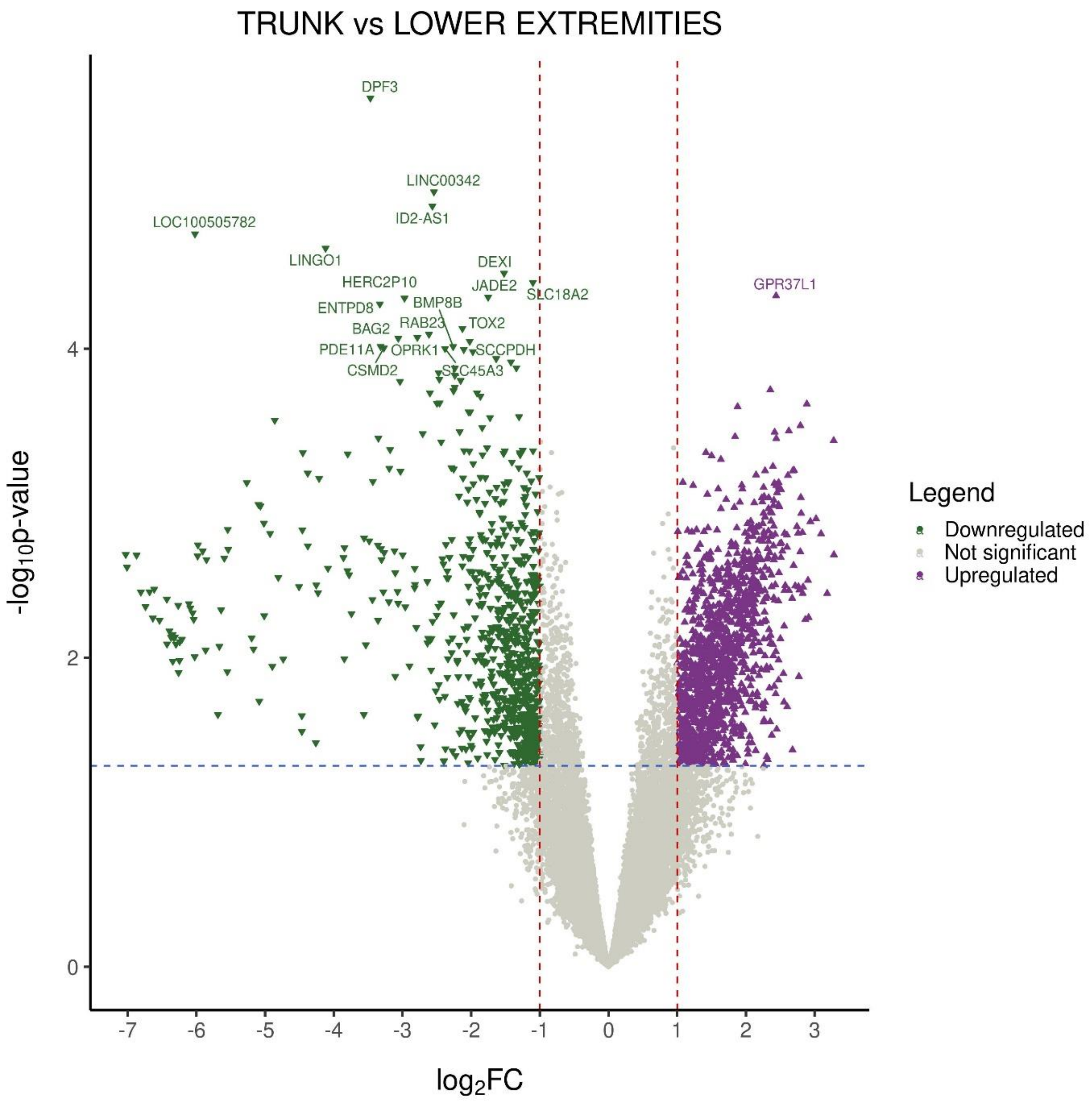

Figure S3: Volcano plot for T vs LL comparison.

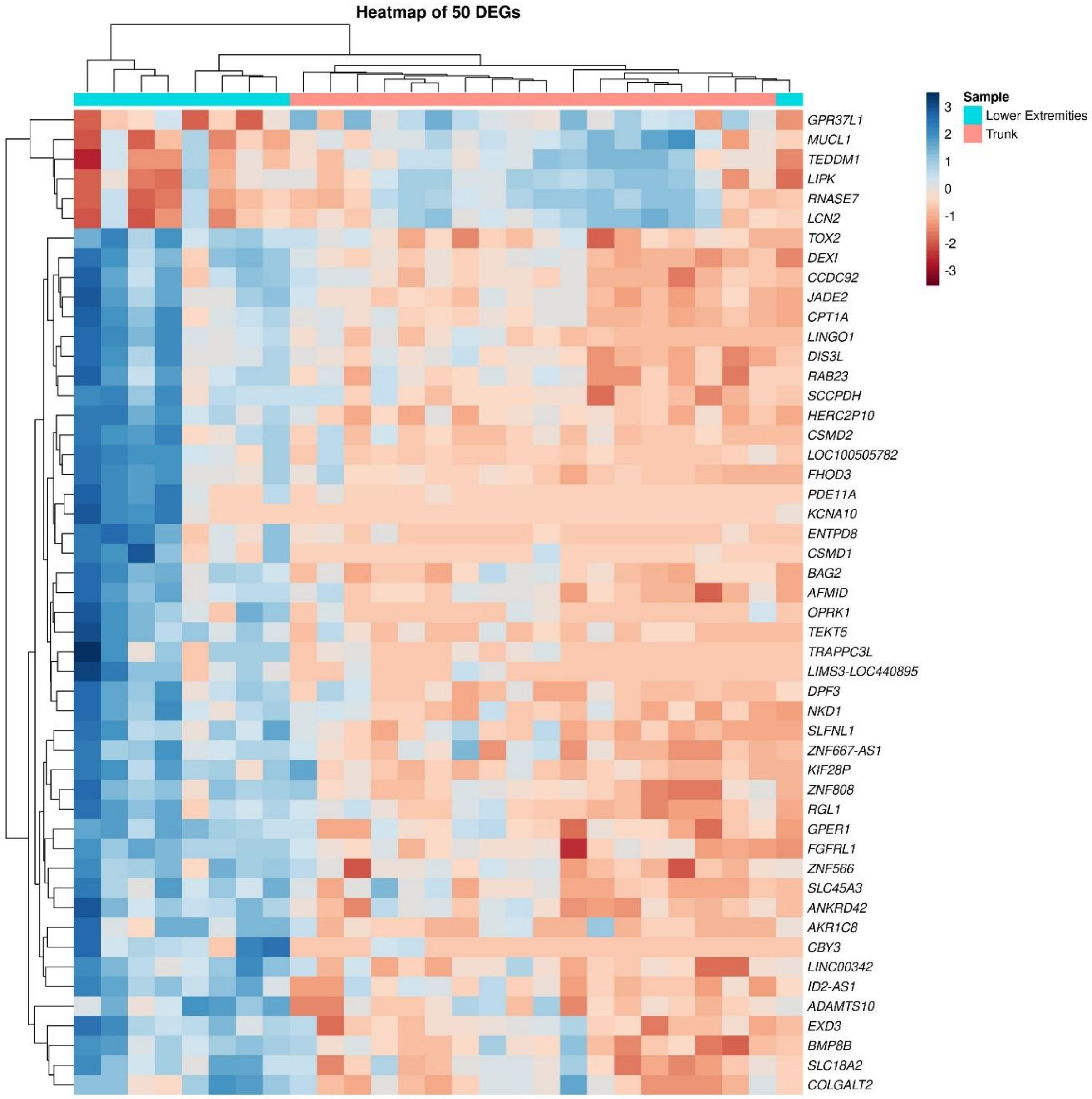

Figure S4: Heatmap for T vs LL comparison.

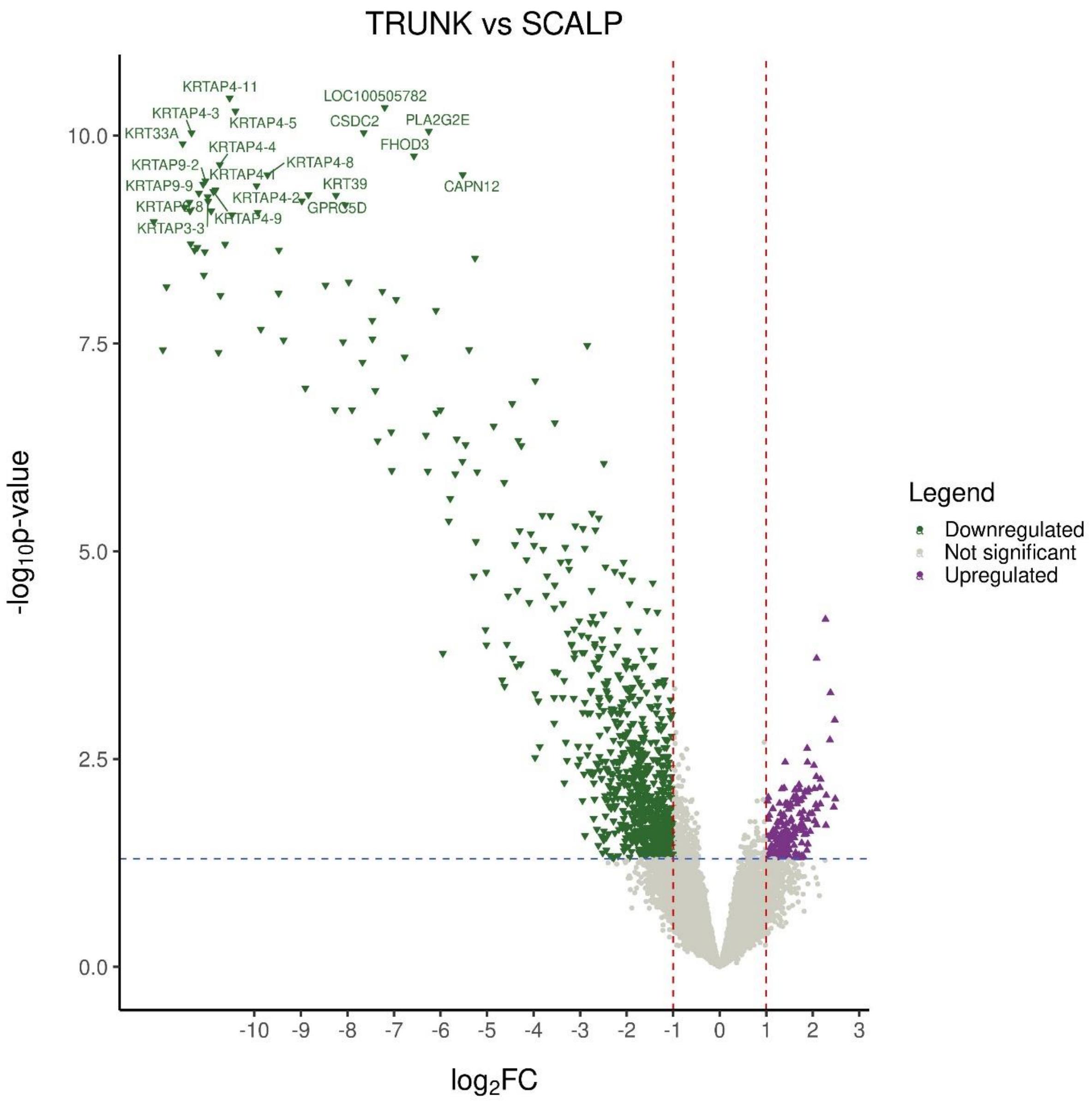

Figure S5: Volcano plot for T vs S comparison.

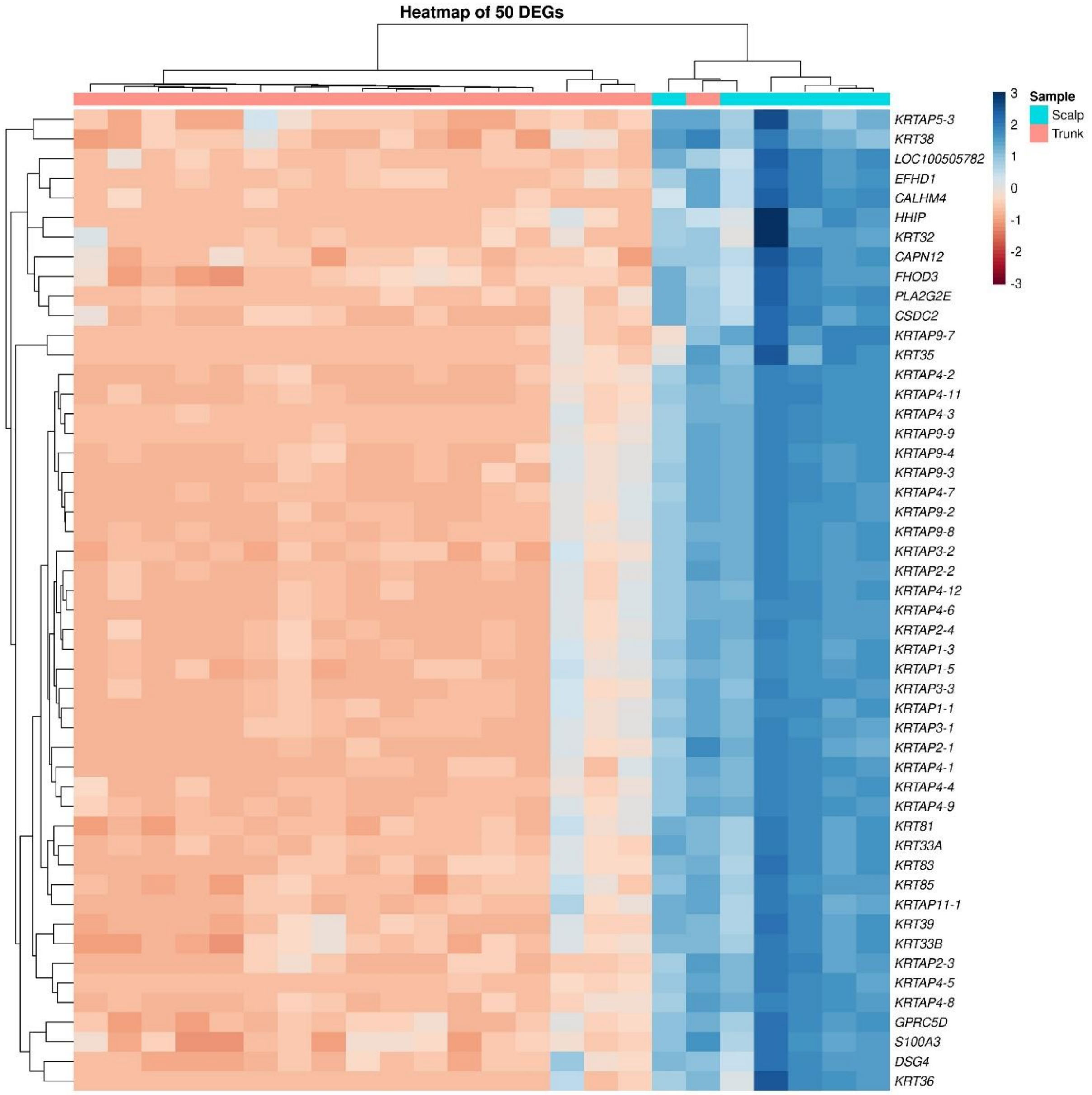

Figure S6: Heatmap for T vs S comparison.

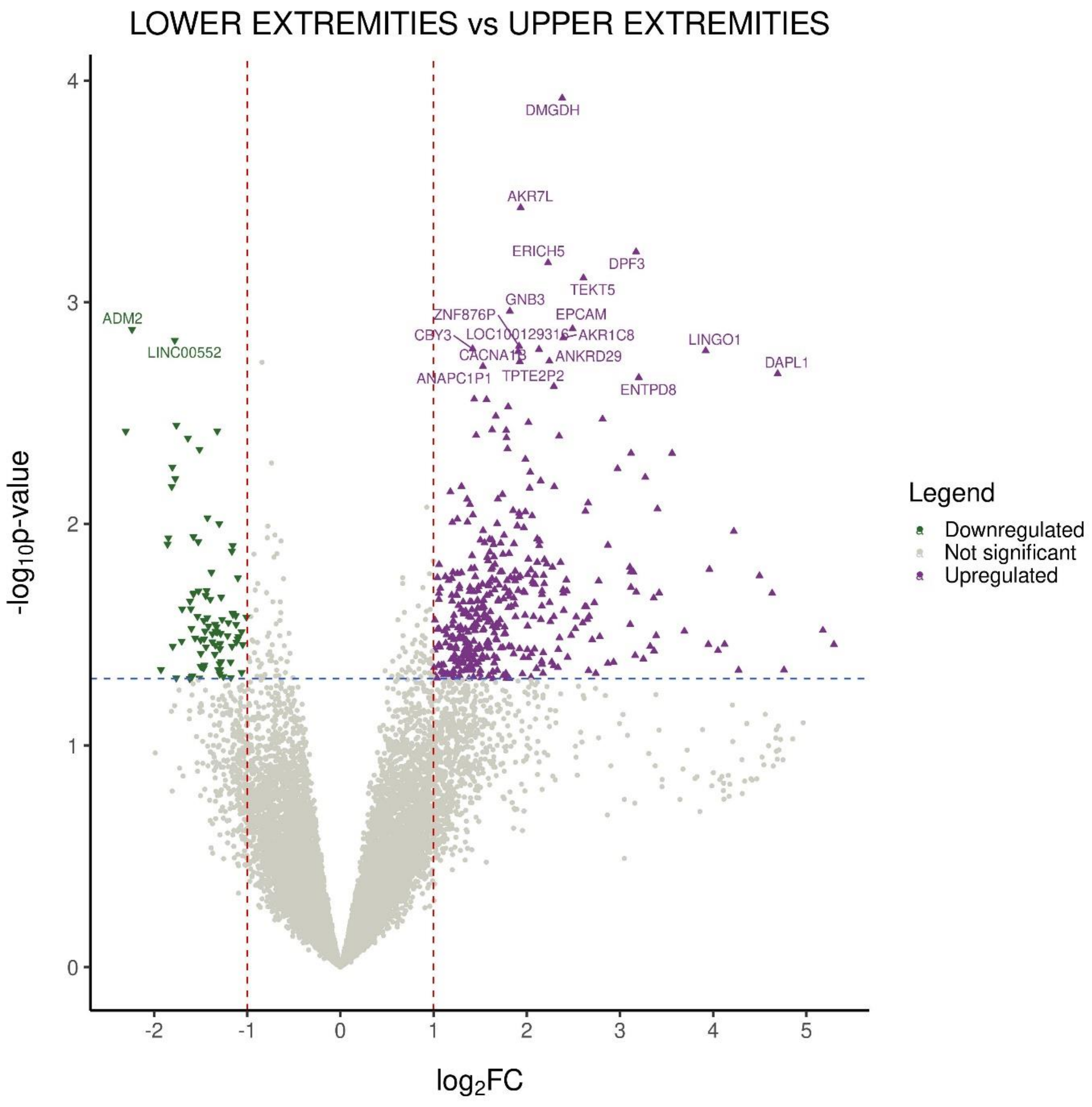

Figure S7: Volcano plot for LL vs UL comparison.

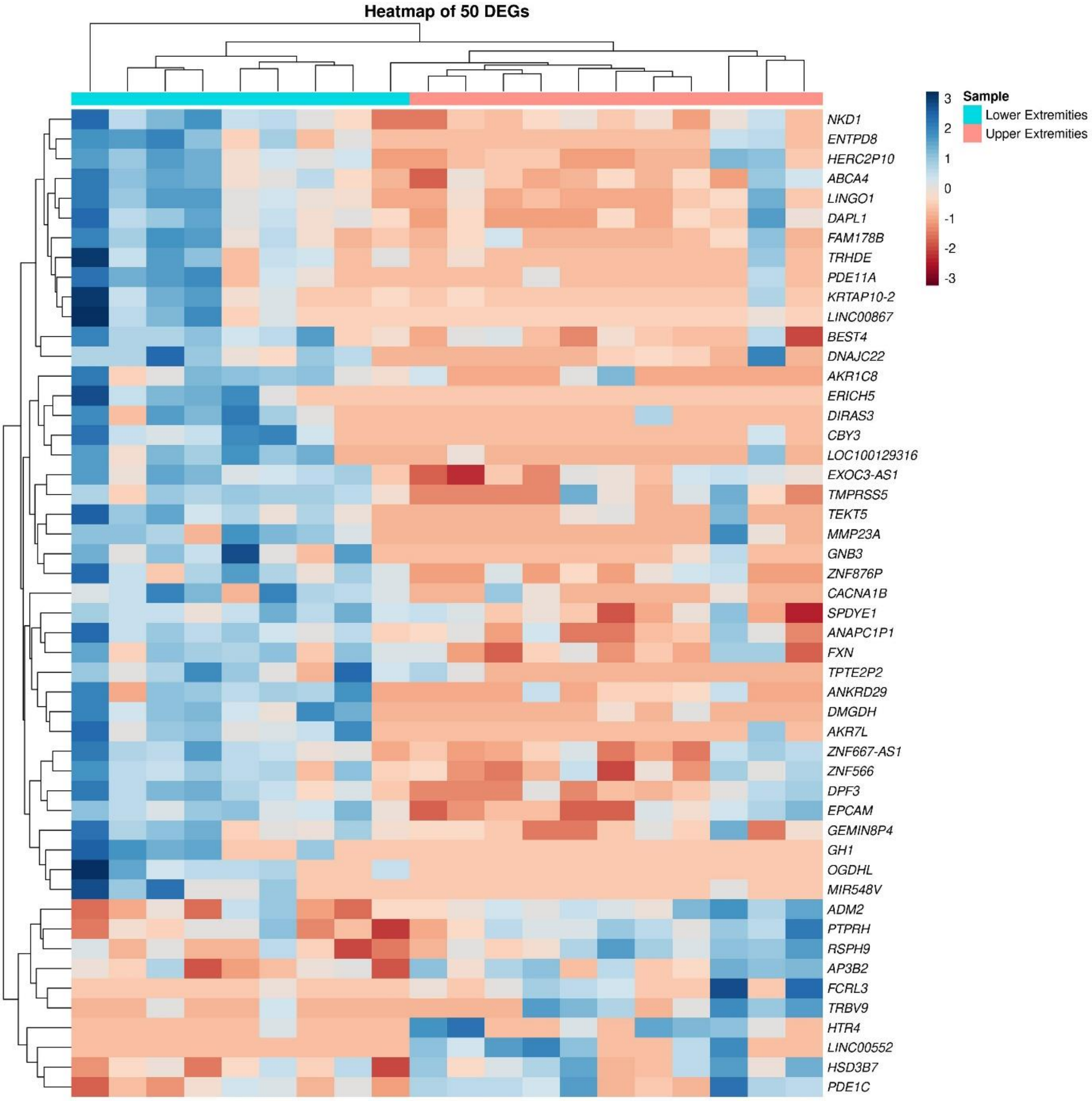

Figure S8: Heatmap for LL vs UL comparison.

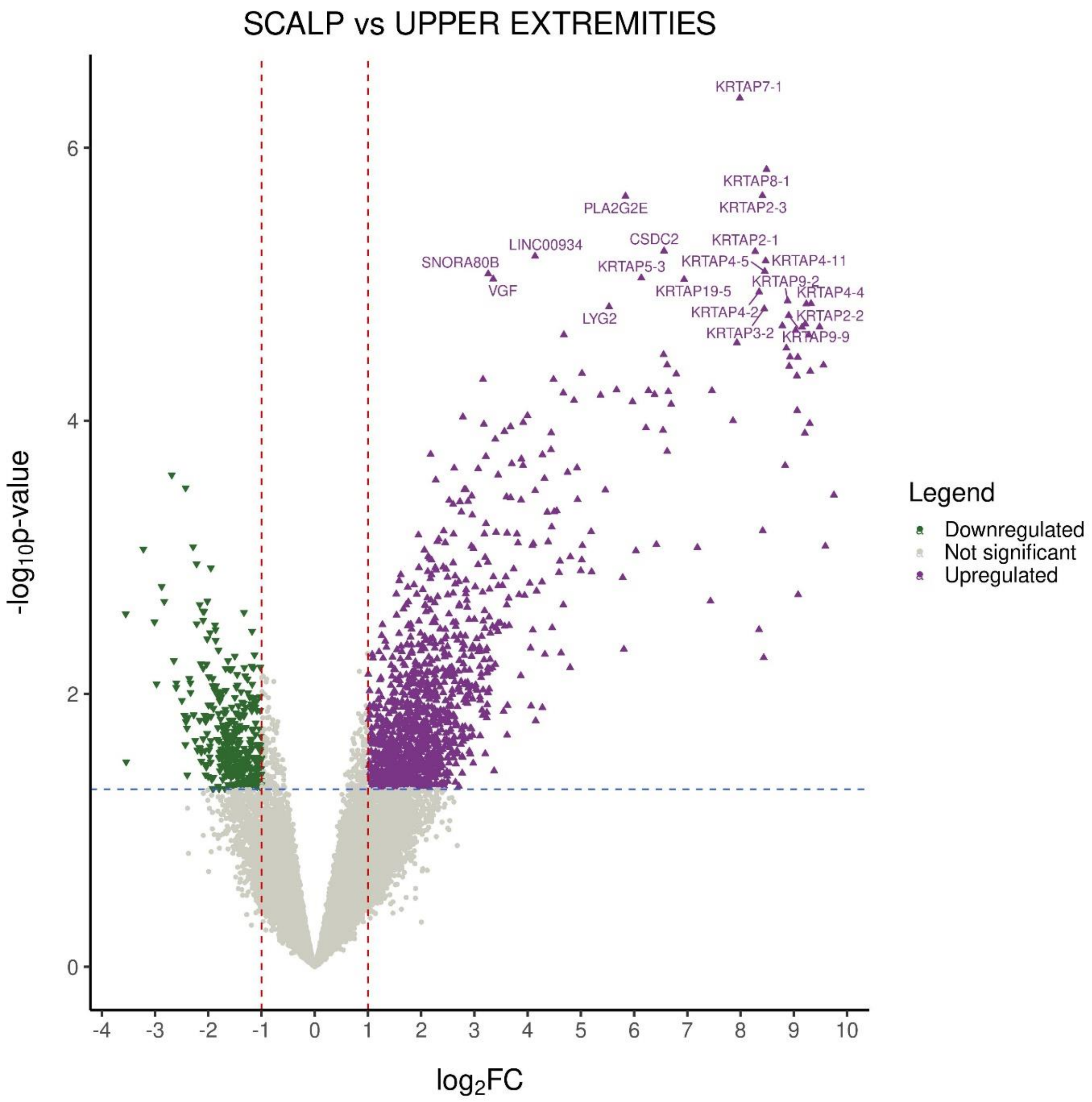

Figure S9: Volcano plot for S vs UL comparison.

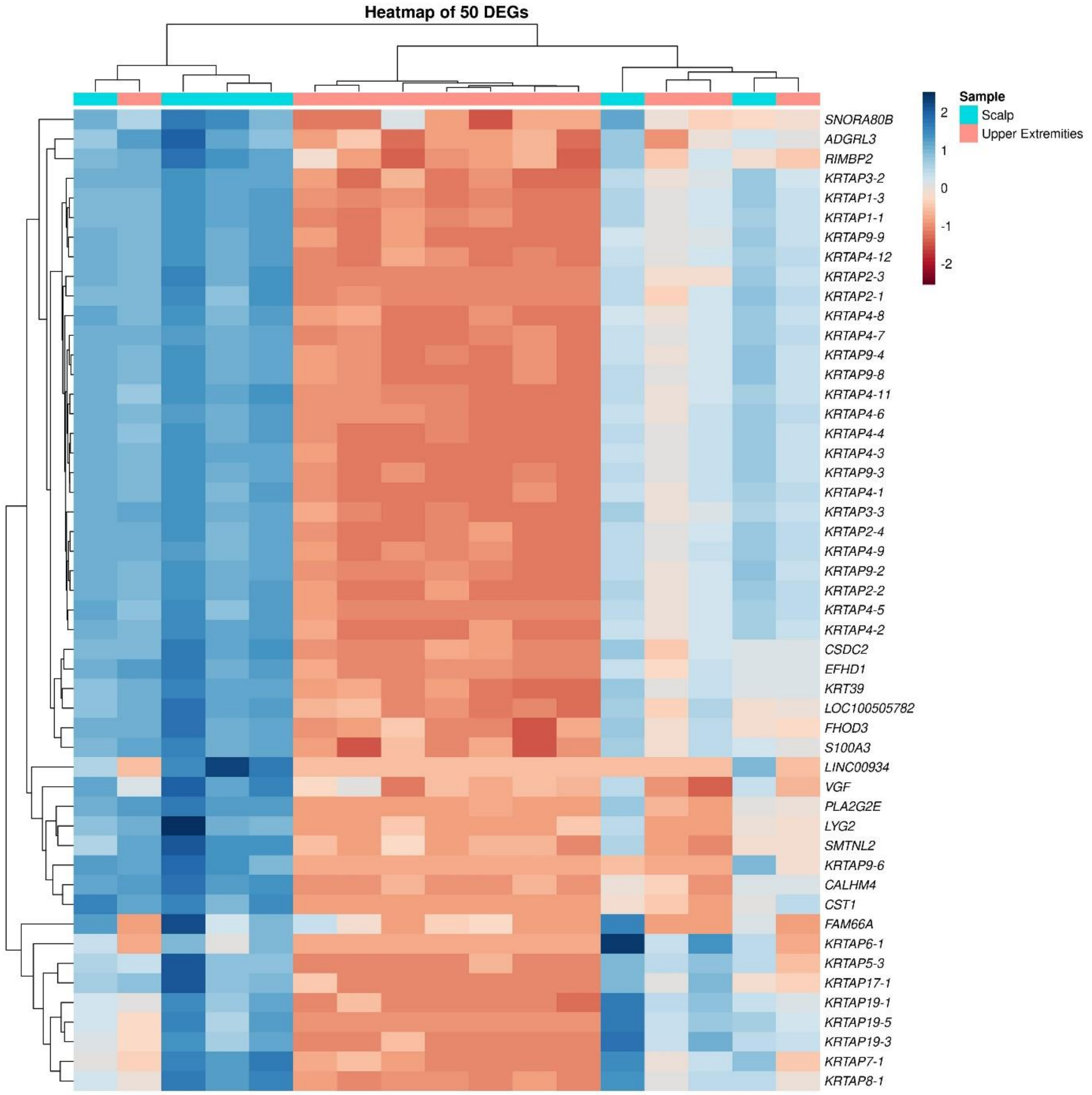

Figure S10: Heatmap for S vs UL comparison.

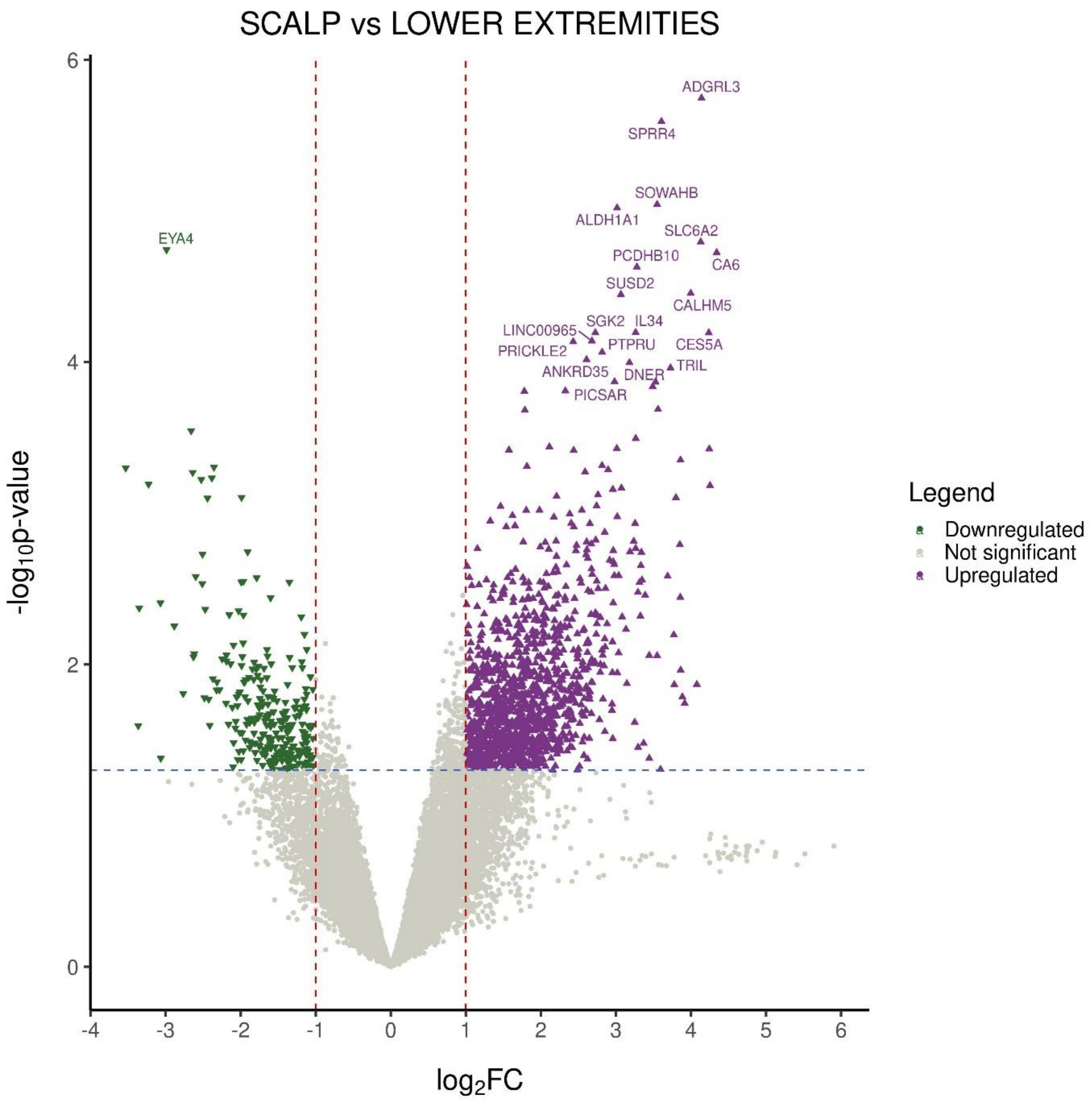

Figure S11: Volcano plot for S vs LL comparison.

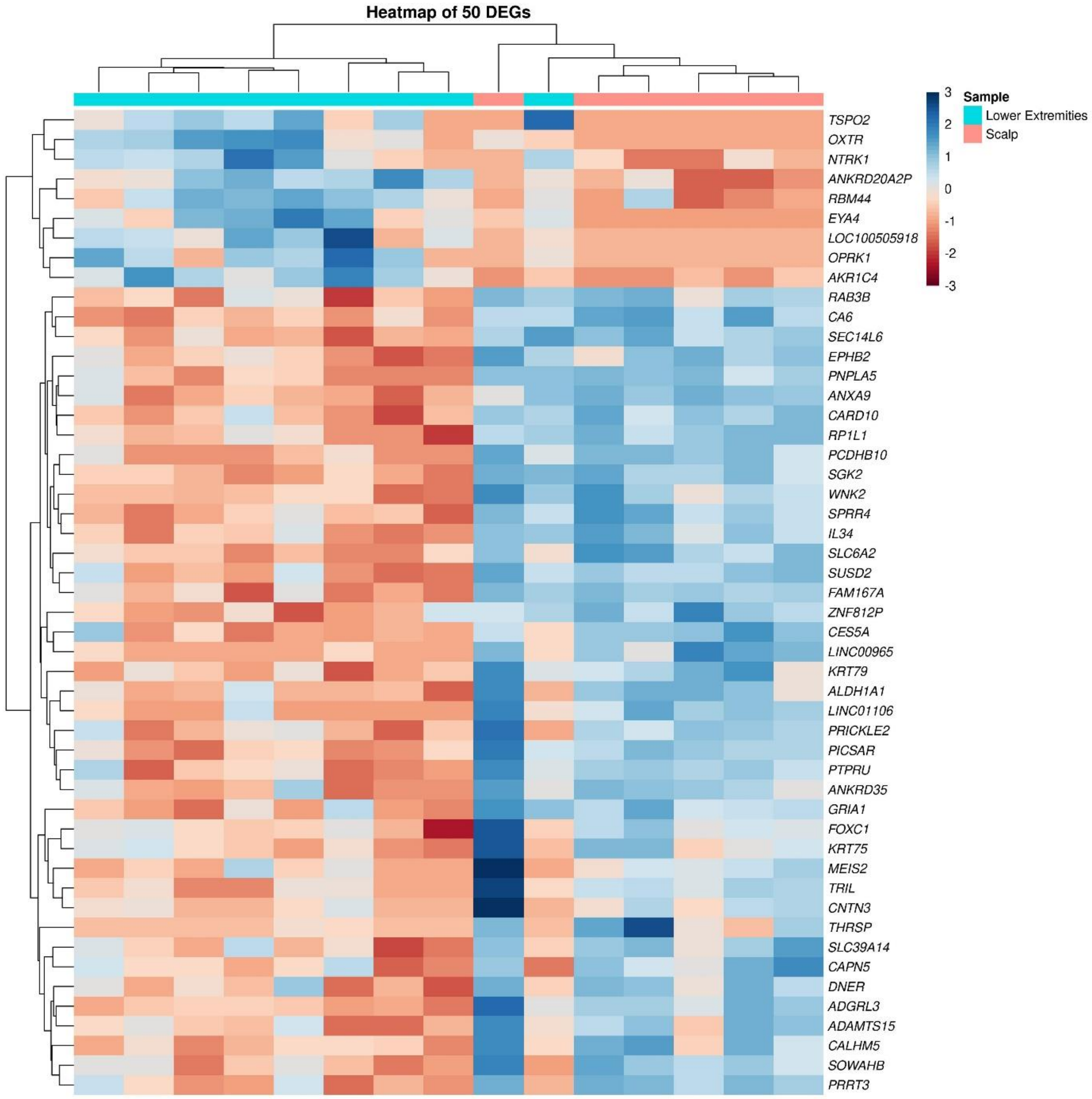

Figure S12: Heatmap for S vs LL comparison.
