# Supplementary material for: Transcriptomic Profiling of Psoriatic Lesions by Tape-Stripping Reveals Site-Specific Differences
Source: J Clin Med. 2026 May 22;15(11):4034. doi: 10.3390/jcm15114034 (PMC13258650; doi:10.3390/jcm15114034)
Supplement: Supplementary file 1 [file jcm-15-04034-s001.zip › Supplementary Figures S13 - S20.pdf]

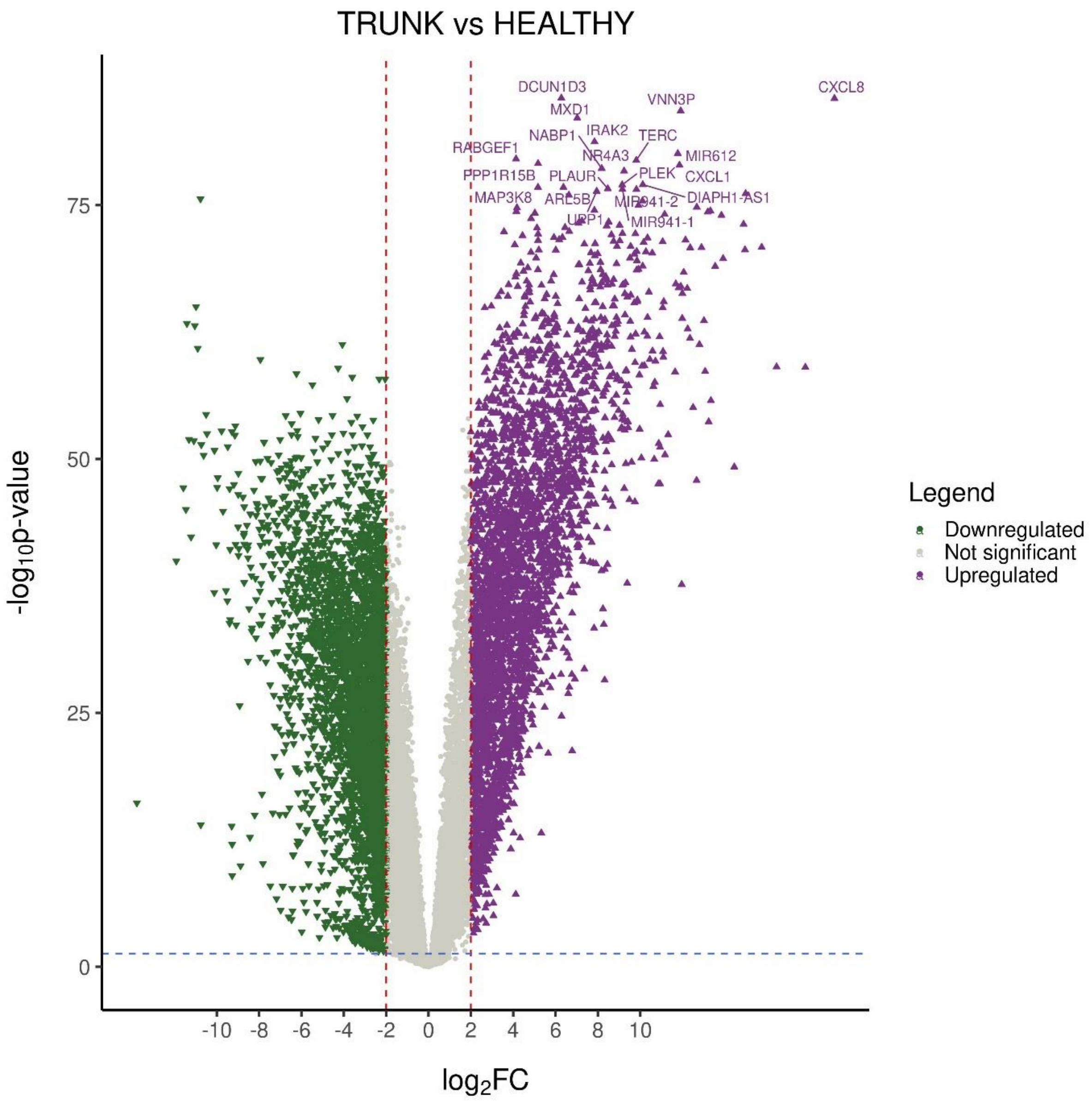

Figure S13: Volcano plot for T vs HC comparison.

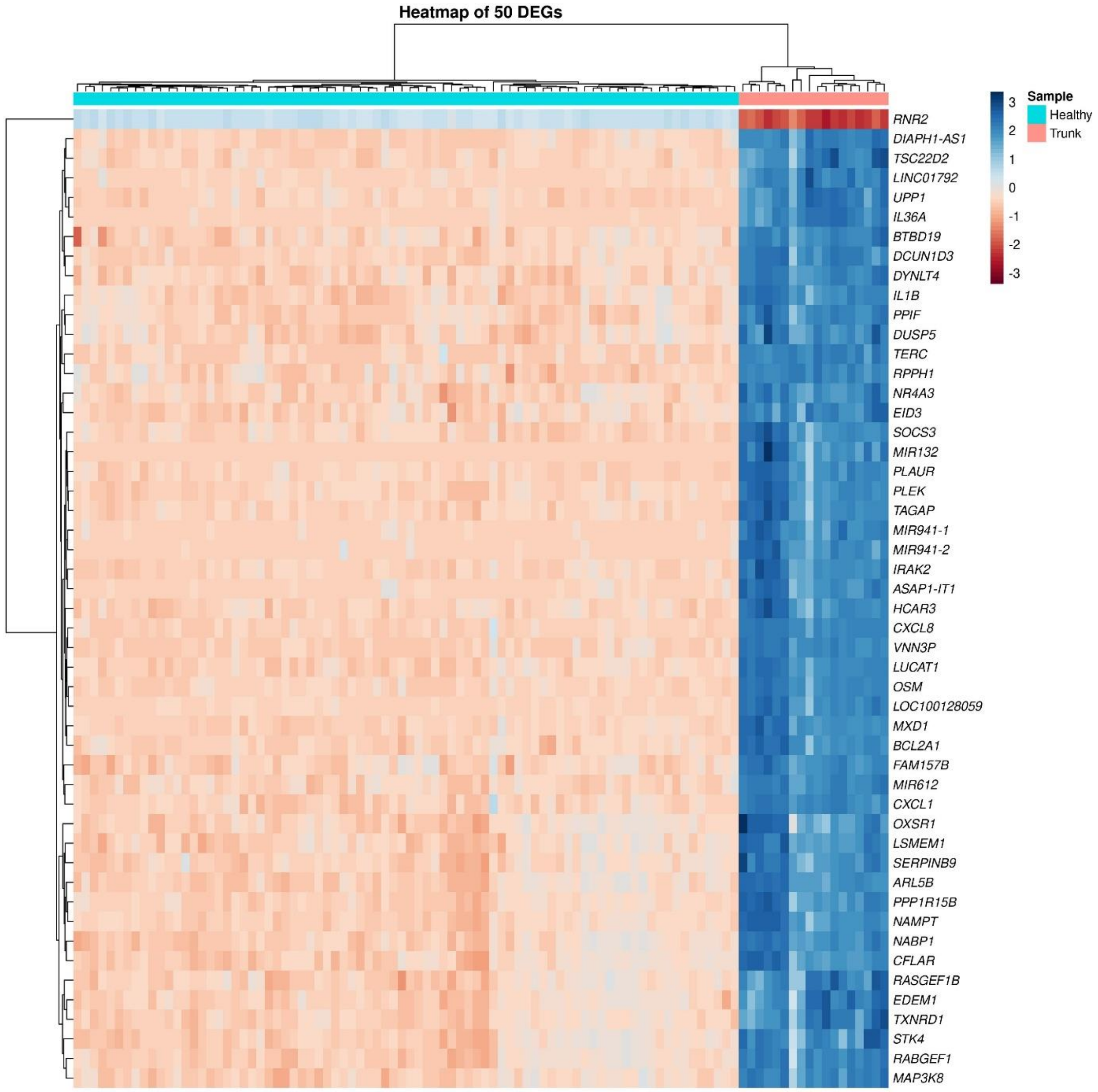

Figure S14: Heatmap for T vs HC comparison.

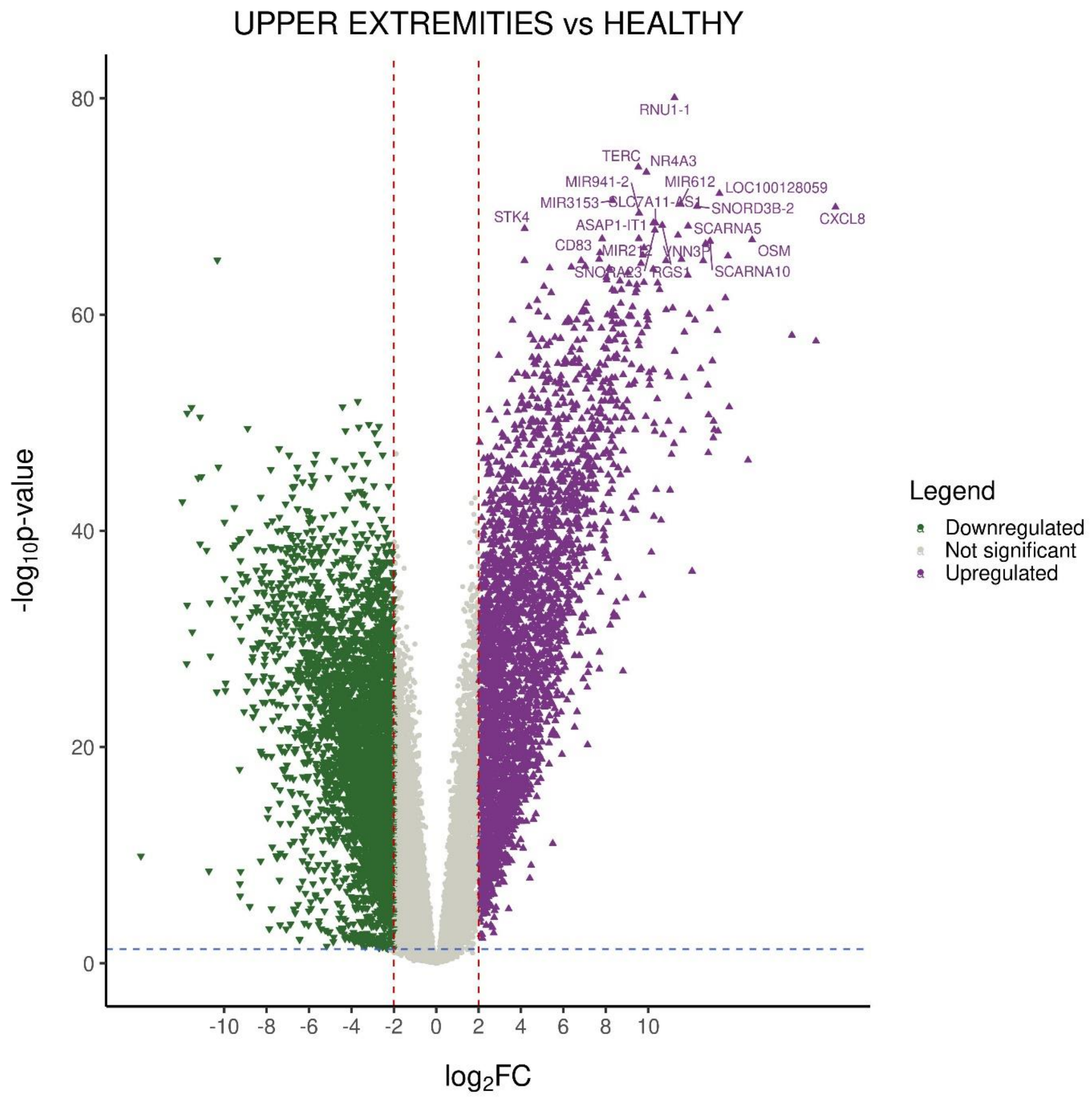

Figure S15: Volcano plot for UL vs HC comparison.

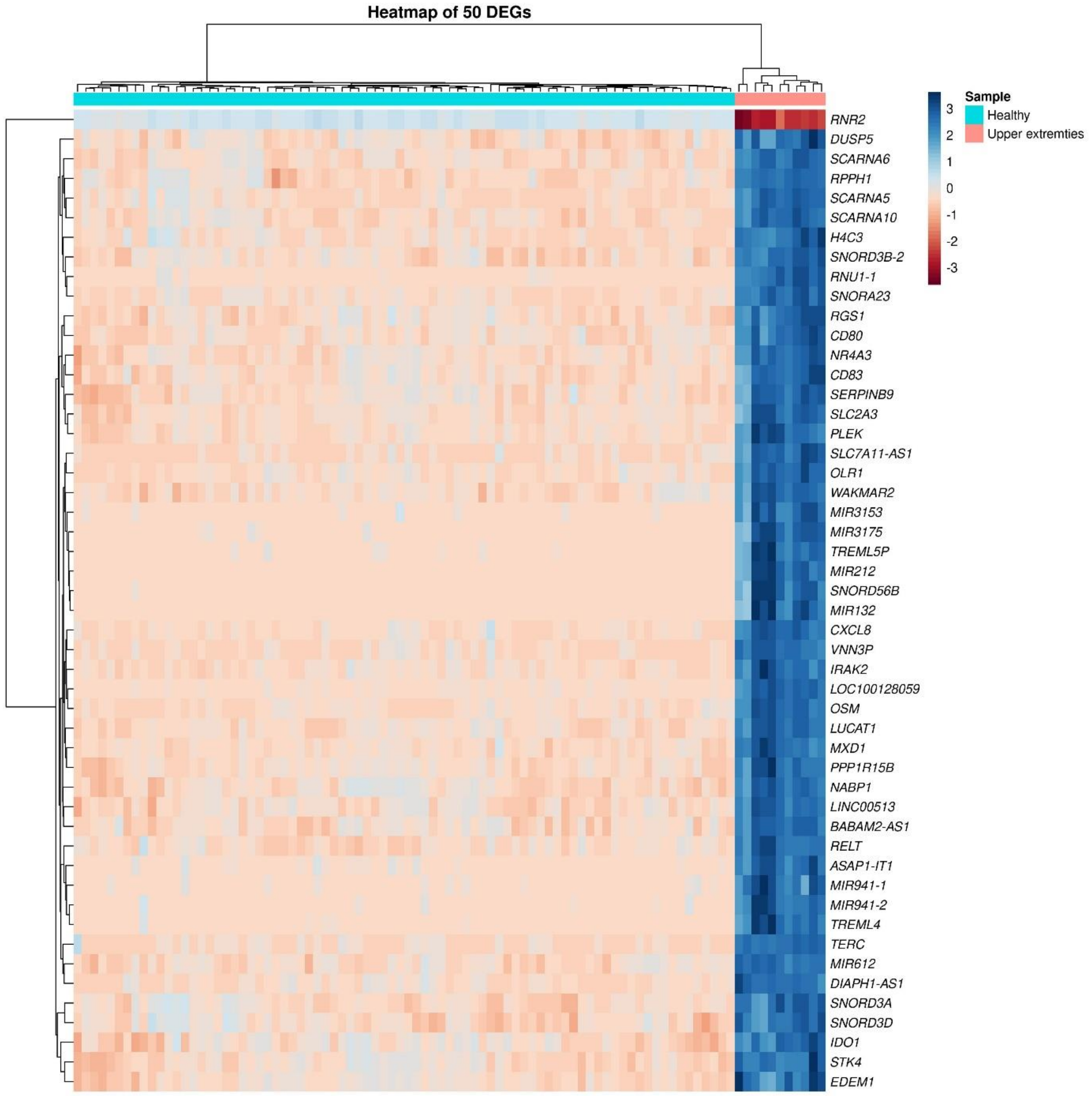

Figure S16: Heatmap for UL vs HC comparison.

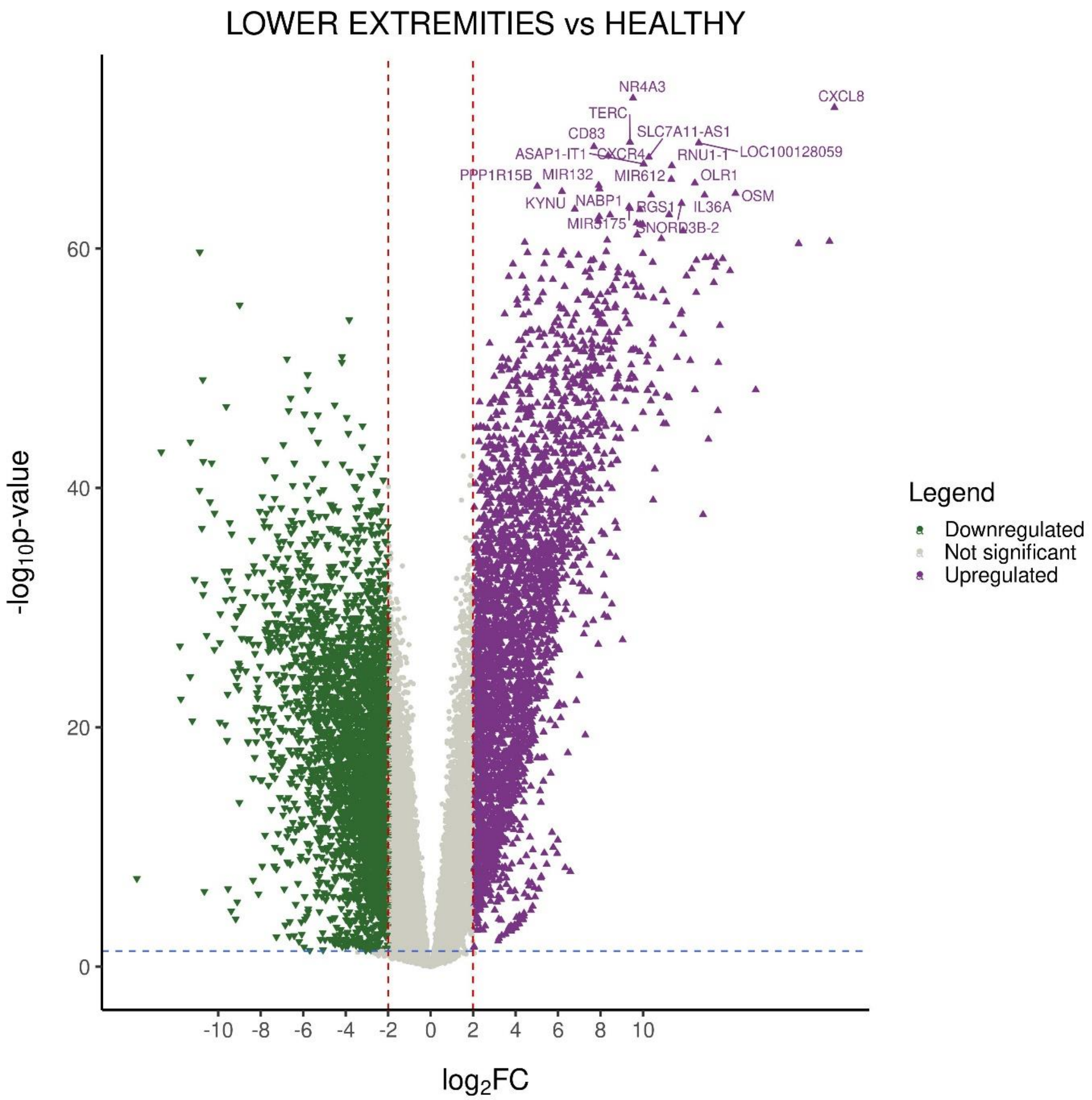

Figure S17: Volcano plot for LL vs HC comparison.

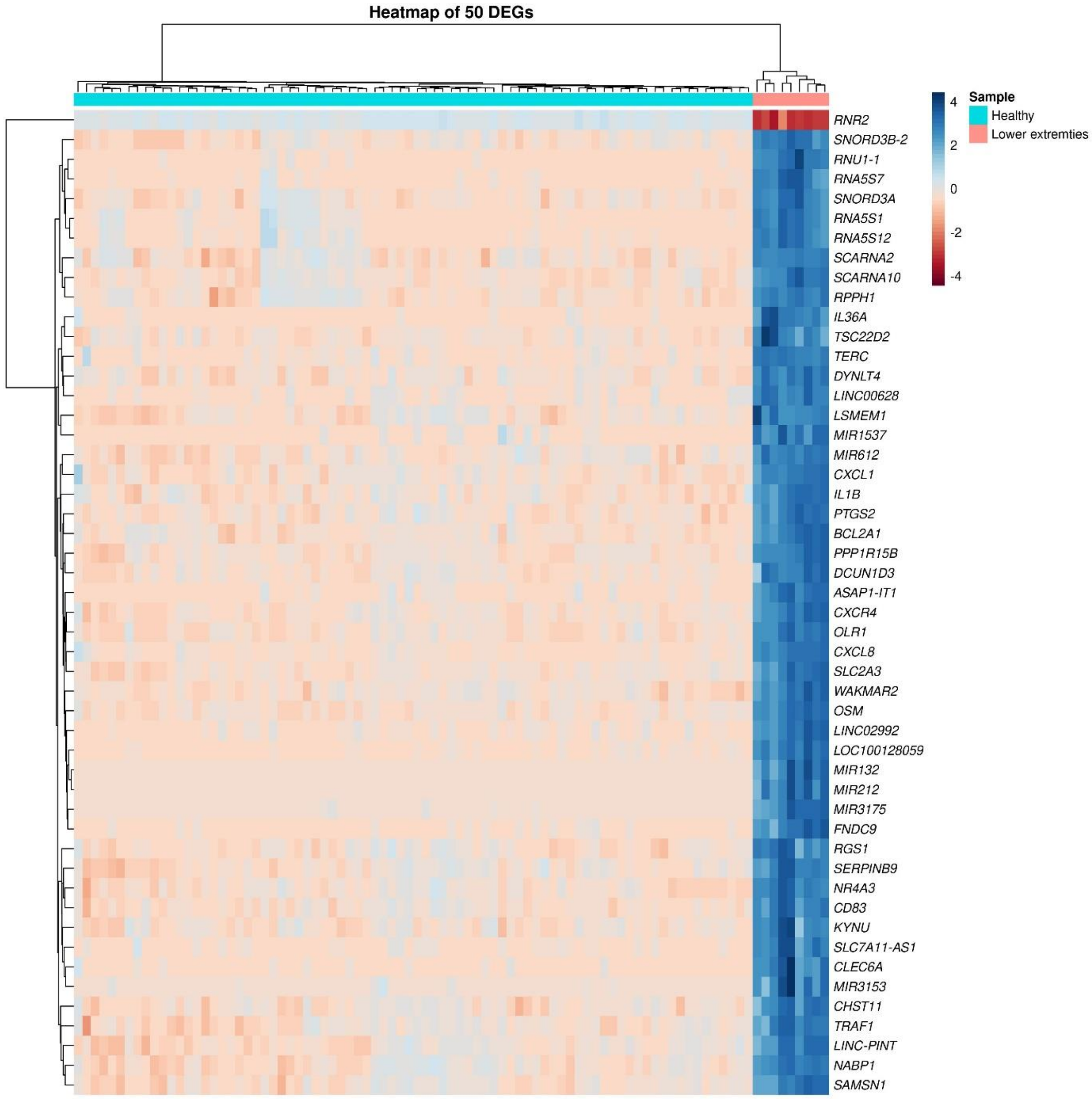

Figure S18: Heatmap for LL vs HC comparison.

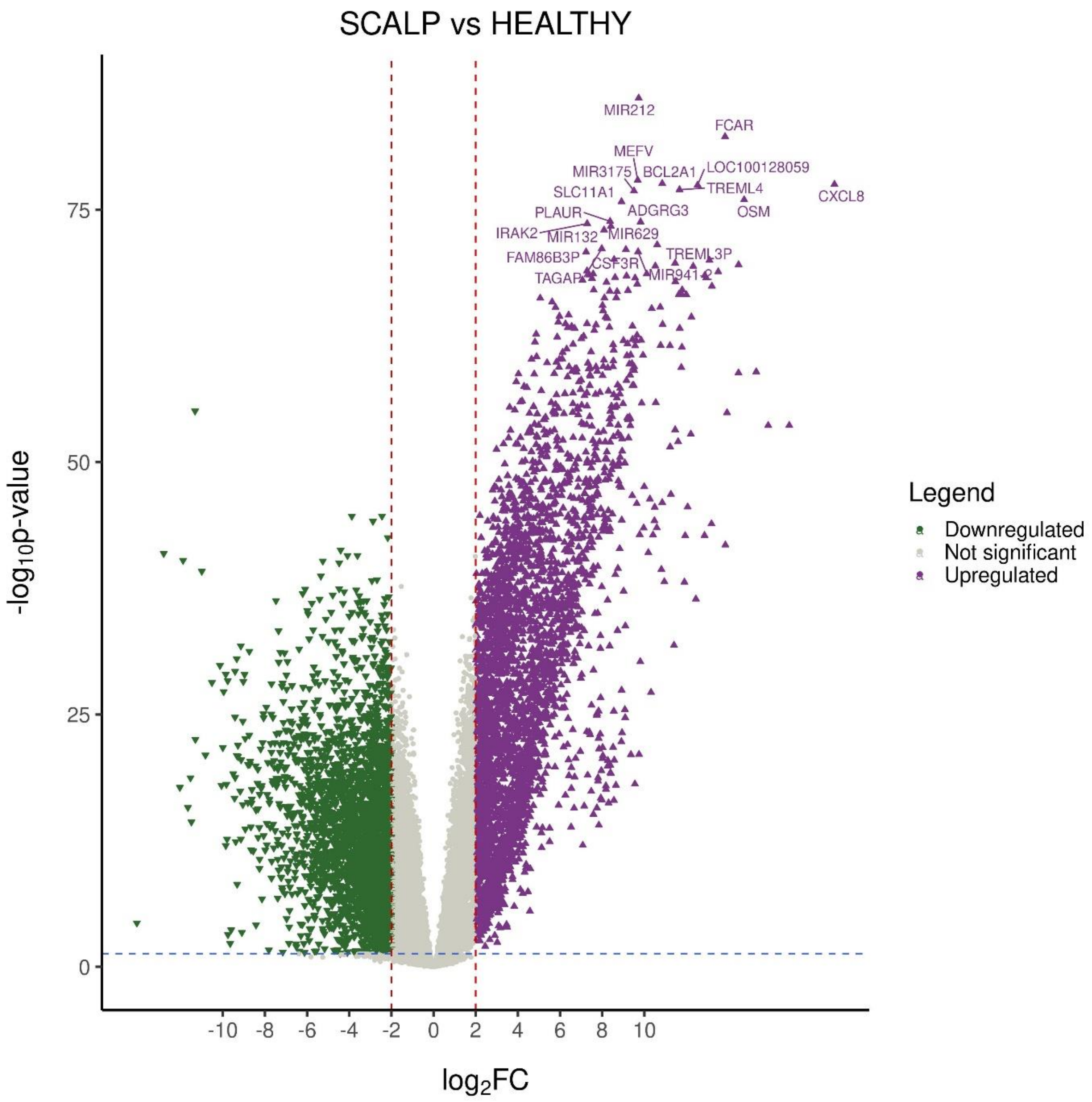

Figure S19: Volcano plot for S vs HC comparison.

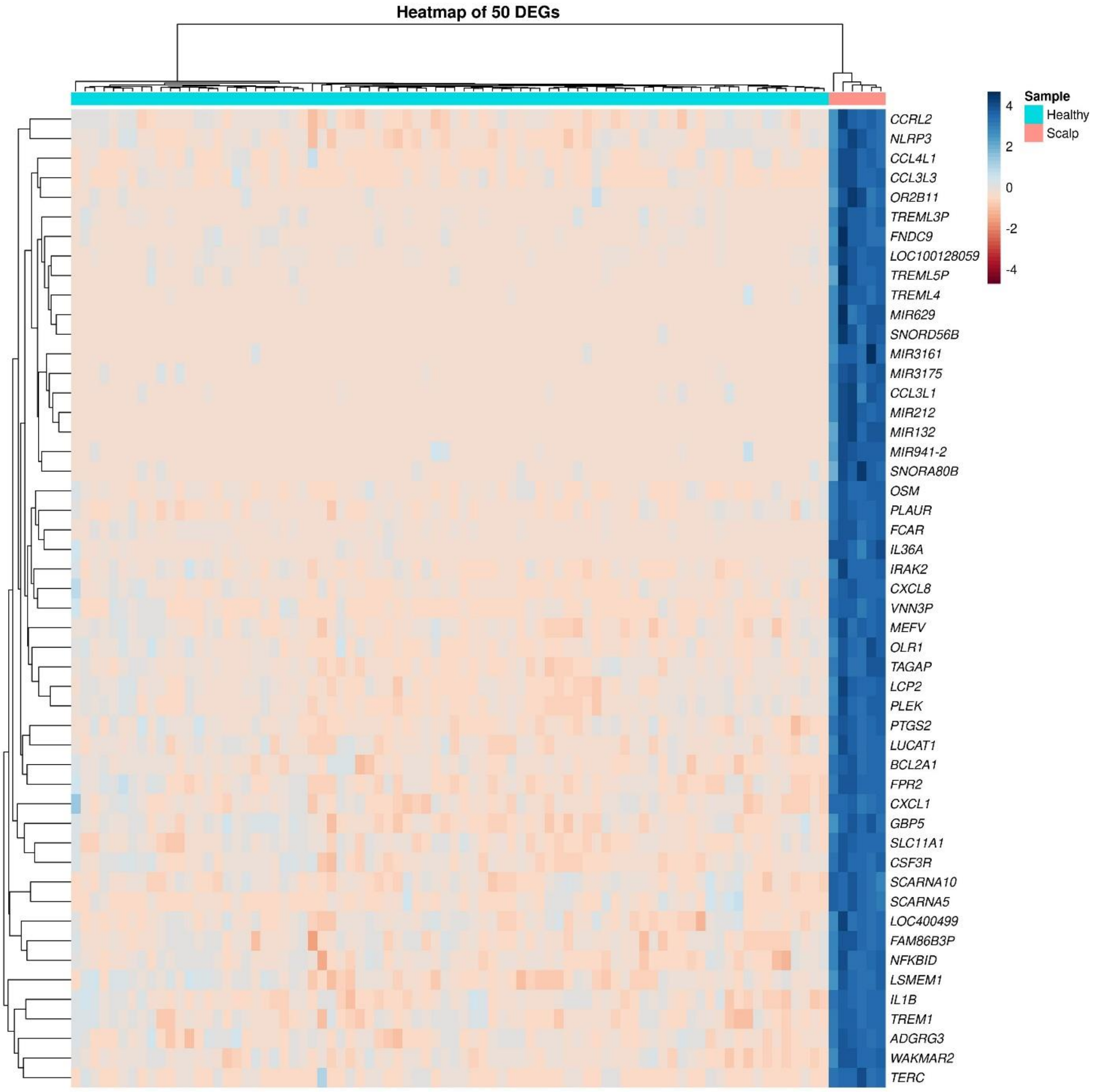

Figure S20: Heatmap for S vs HC comparison.
