# Supplementary material for: Transcriptomic Profiling of Psoriatic Lesions by Tape-Stripping Reveals Site-Specific Differences
Source: J Clin Med. 2026 May 22;15(11):4034. doi: 10.3390/jcm15114034 (PMC13258650; doi:10.3390/jcm15114034)
Supplement: Supplementary file 1 [file jcm-15-04034-s001.zip › Supplementary Figures S21 - S32.pdf]

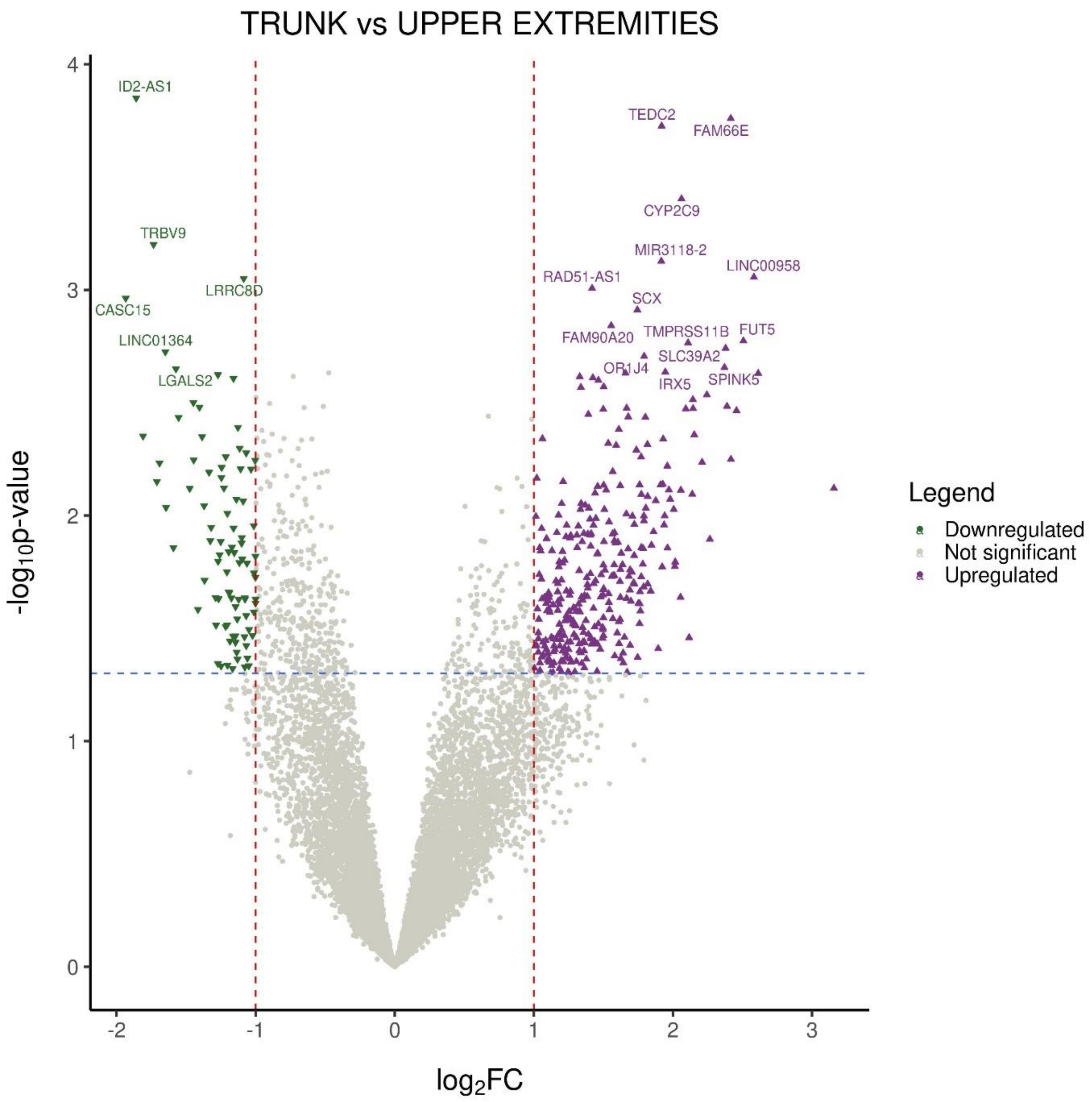

Figure S21: Volcano plot for T vs UL comparison.

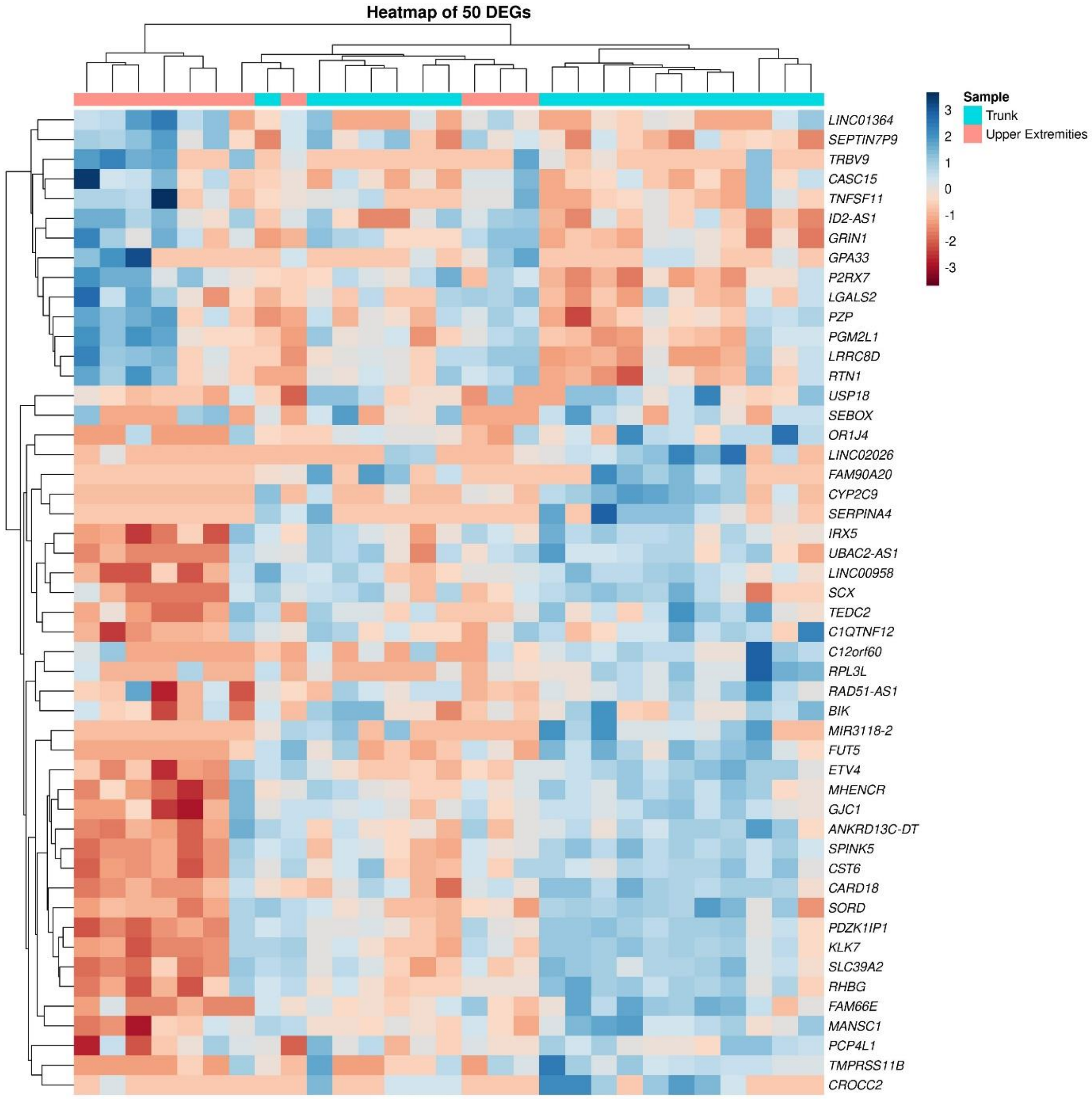

Figure S22: Heatmap for T vs UL comparison.

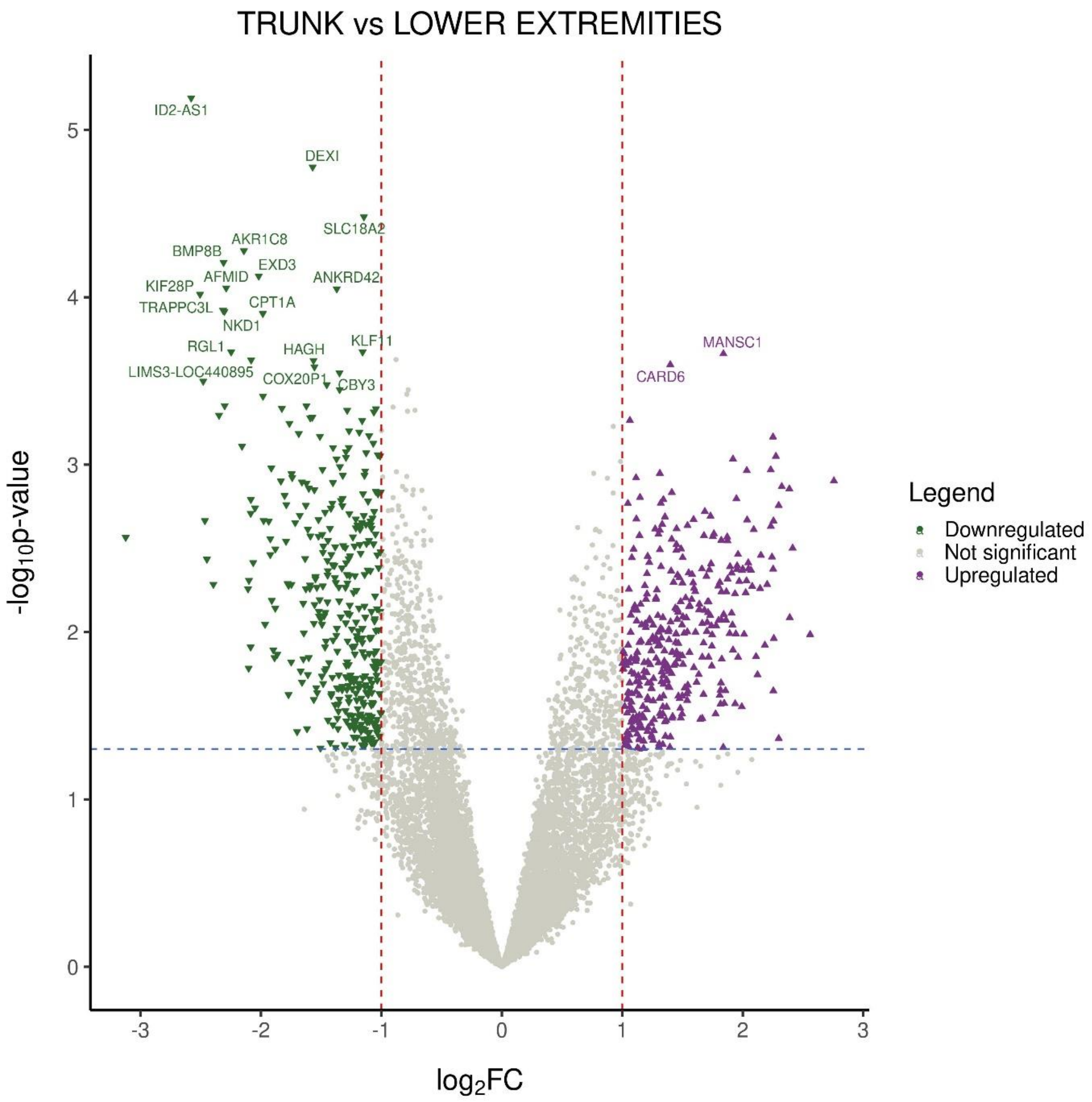

Figure S23: Volcano plot for T vs LL comparison.

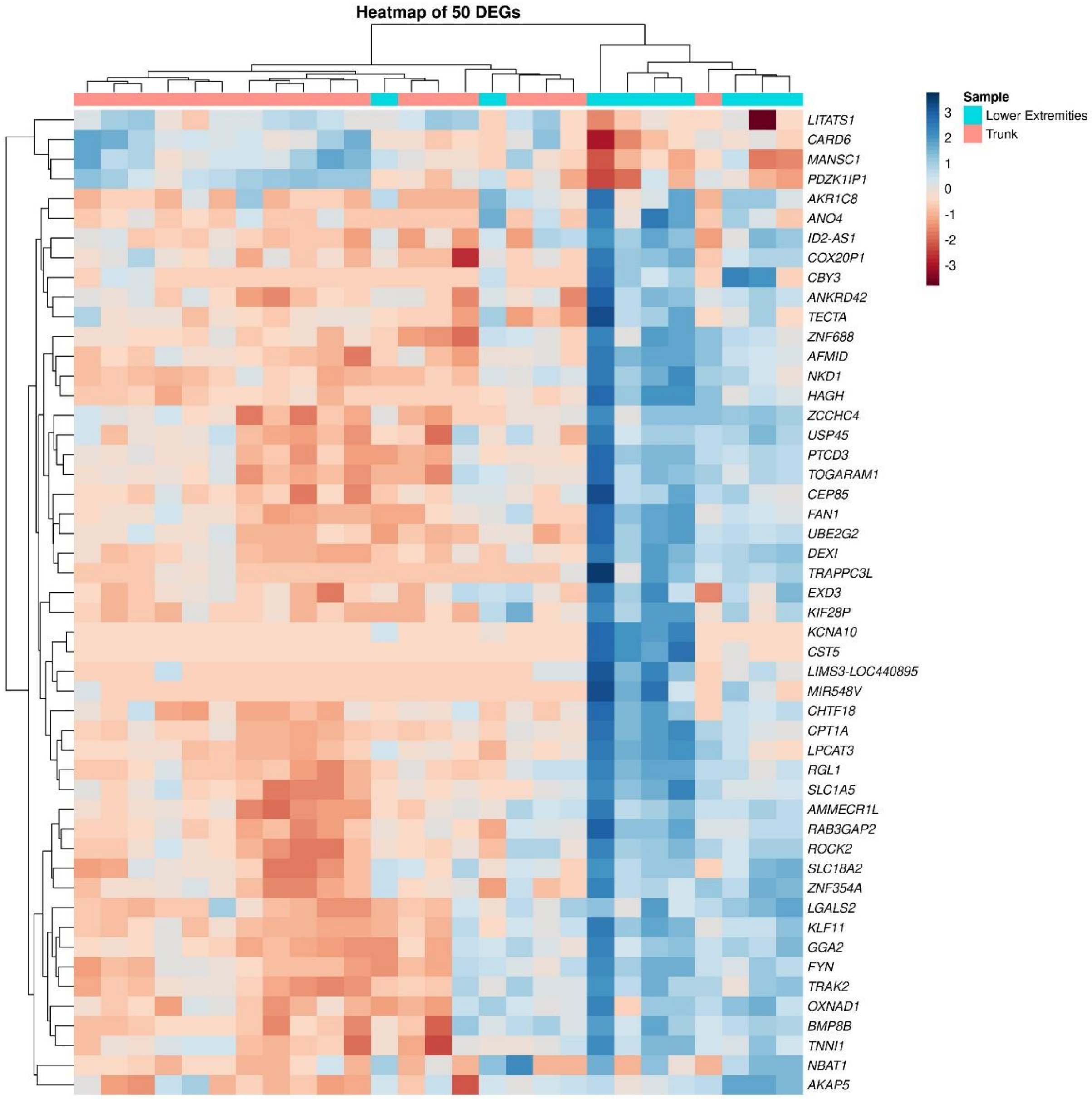

Figure S24: Heatmap for T vs LL comparison.

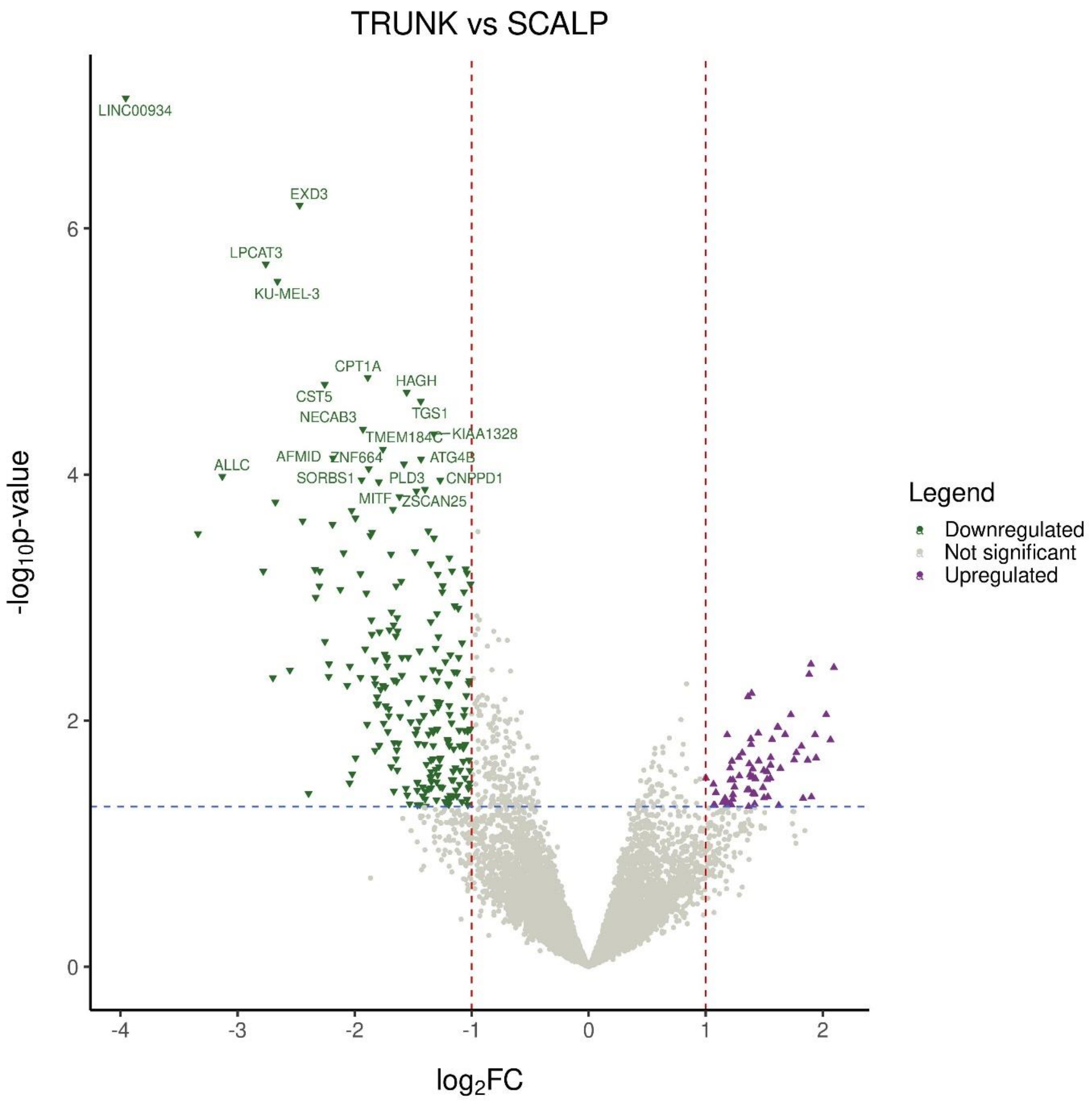

Figure S25: Volcano plot for T vs S comparison.

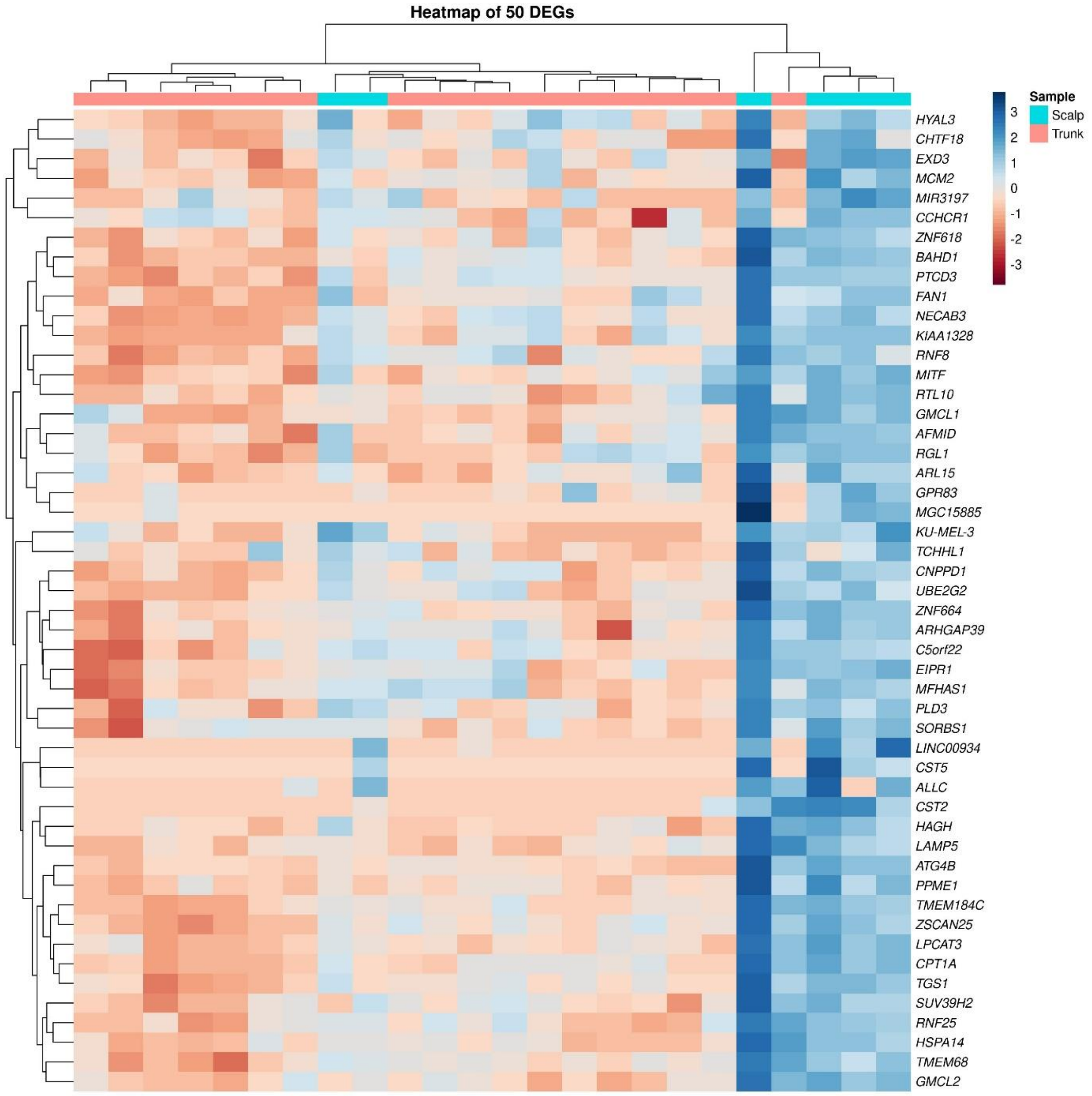

Figure S26: Heatmap for T vs S comparison.

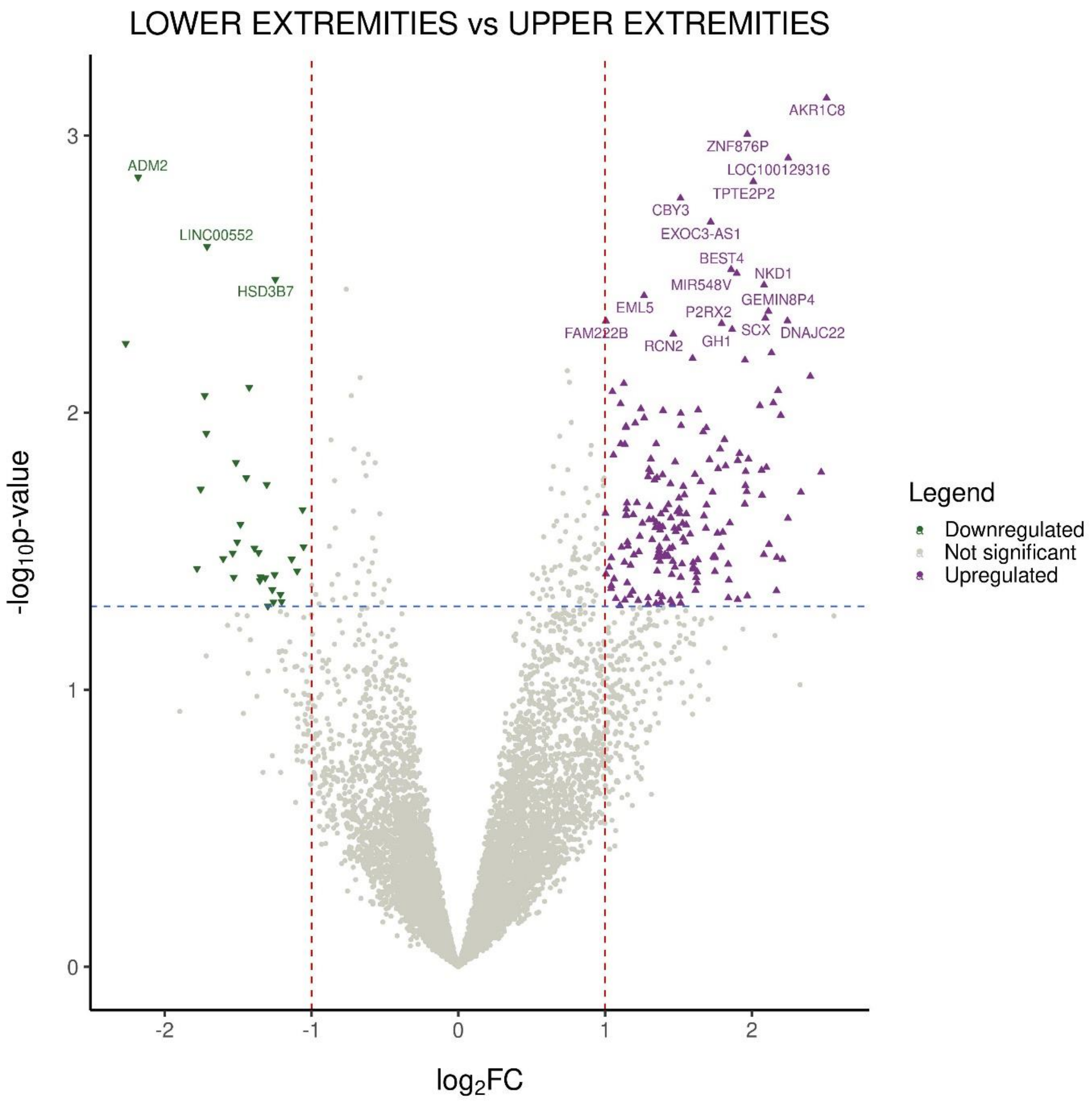

Figure S27: Volcano plot for LL vs UL comparison.

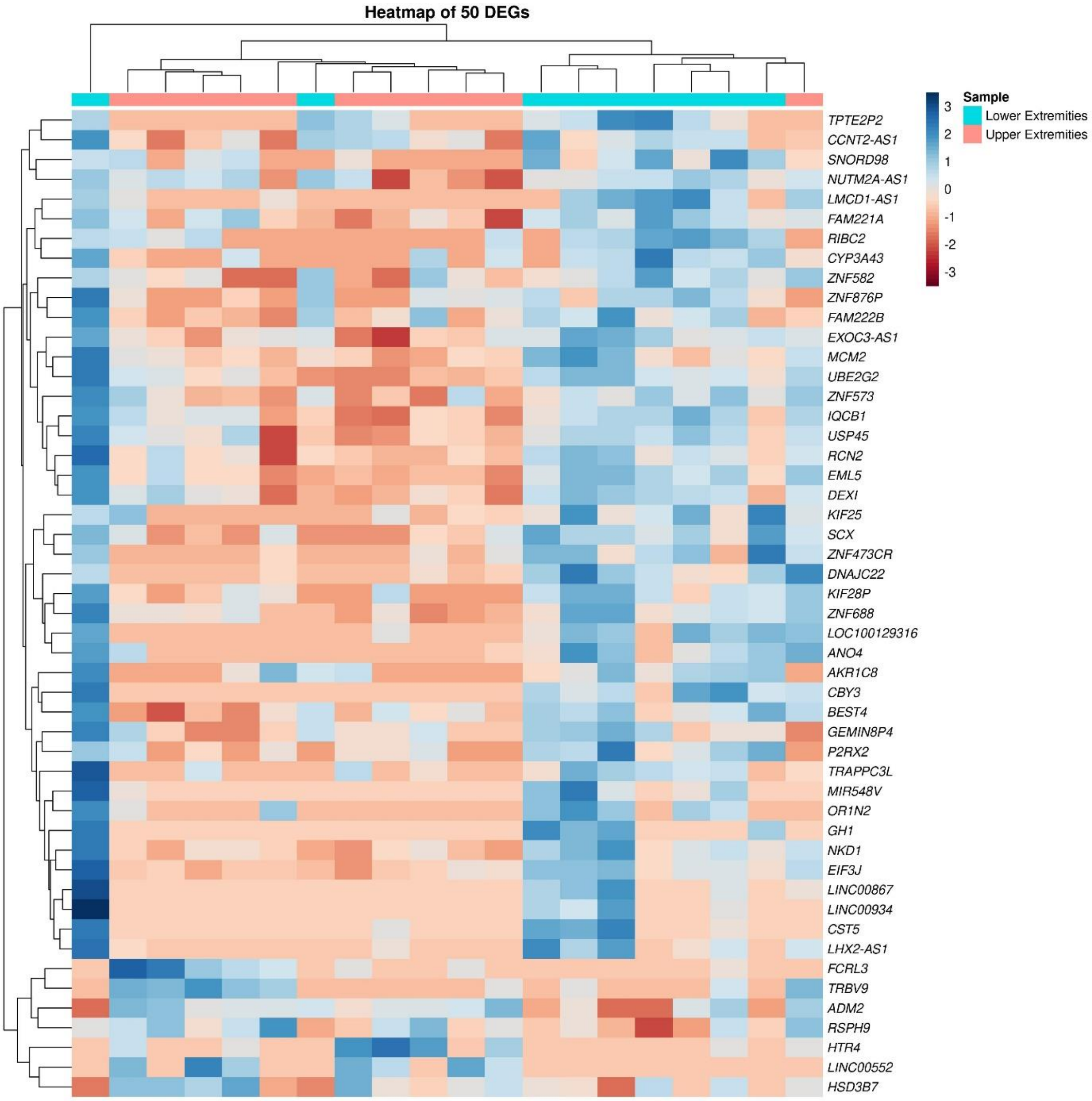

Figure S28: Heatmap for LL vs UL comparison.

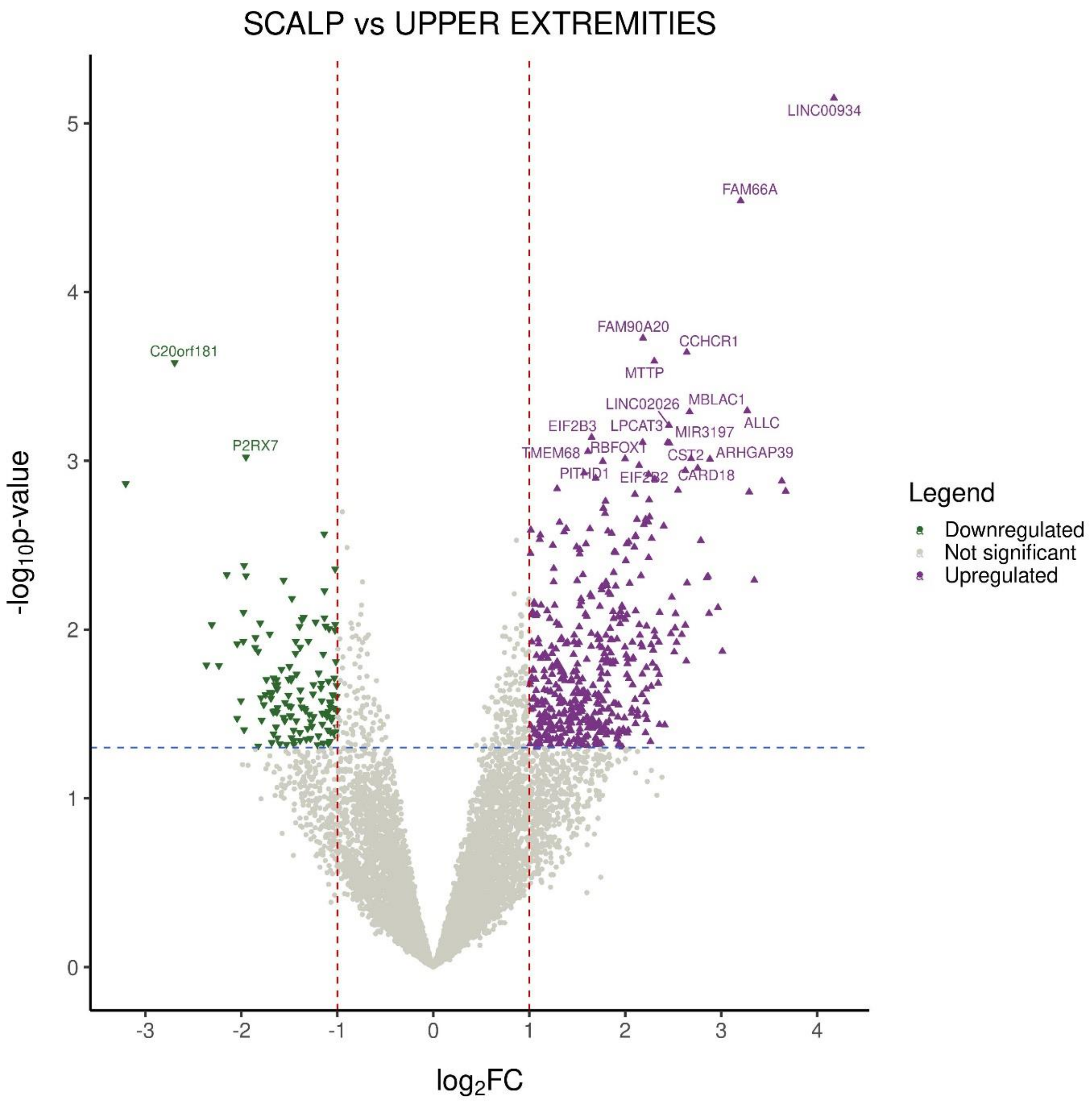

Figure S29: Volcano plot for S vs UL comparison.

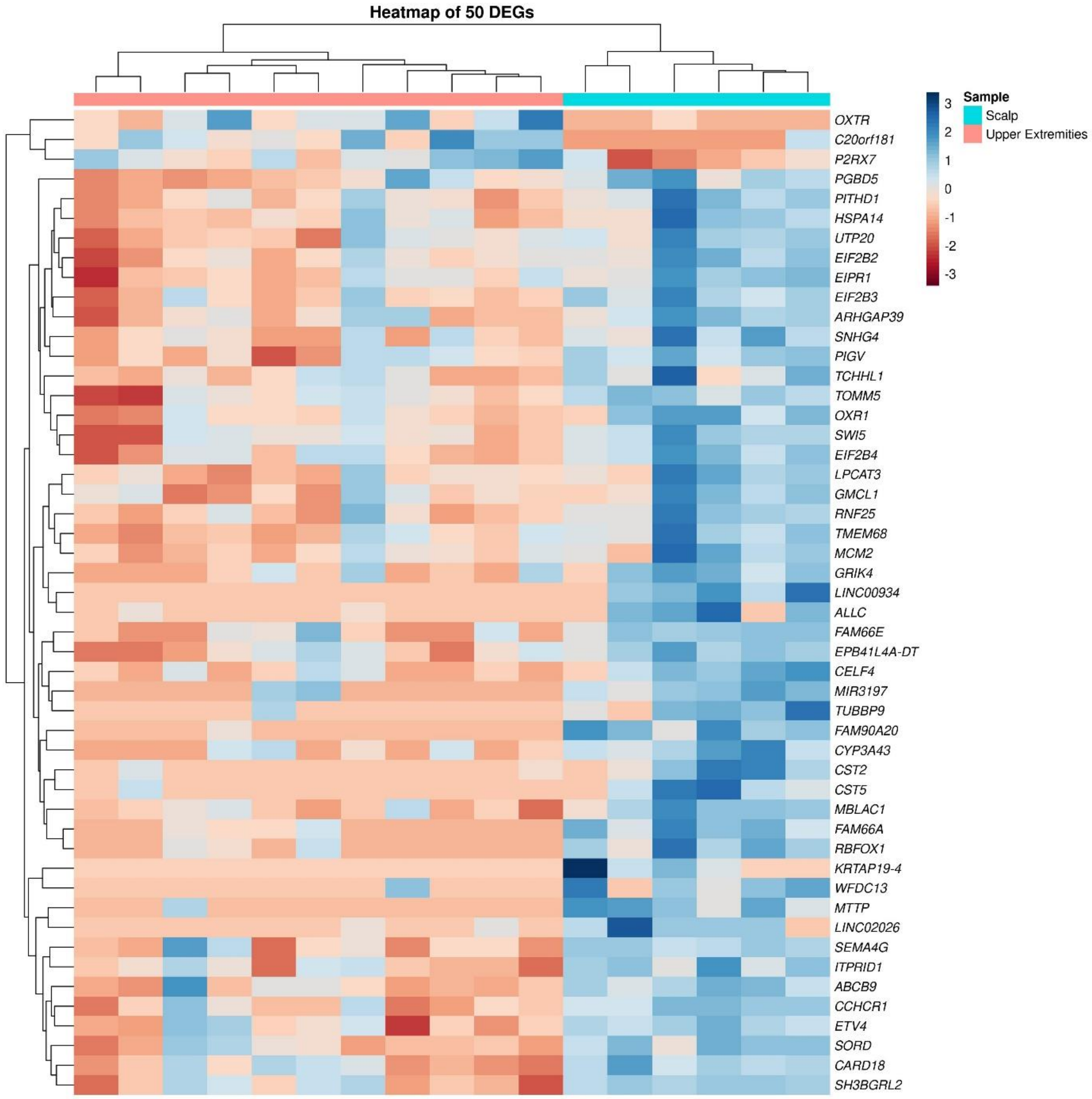

Figure S30: Heatmap for S vs UL comparison.

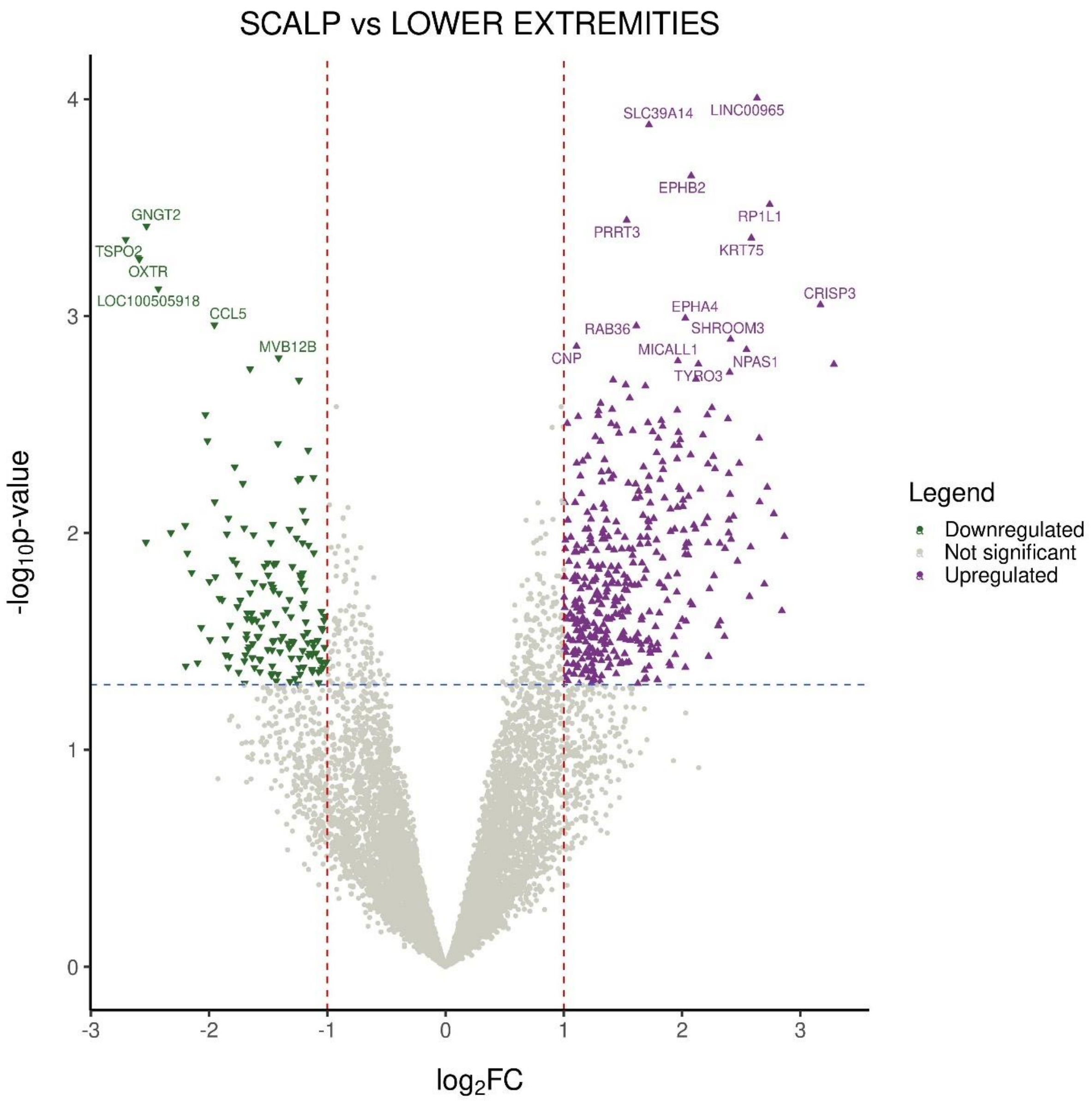

Figure S31: Volcano plot for S vs LL comparison.

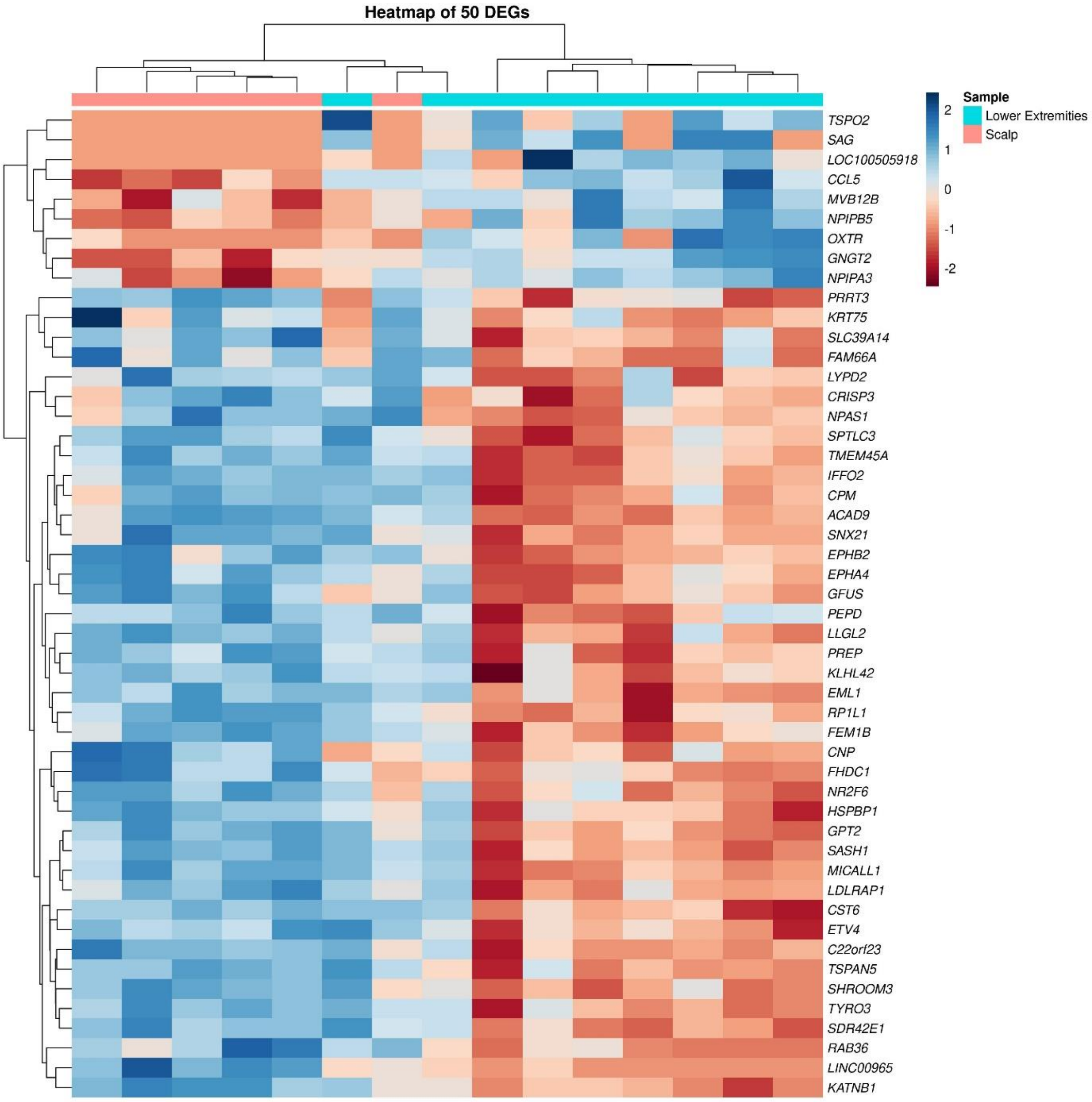

Figure S32: Heatmap for S vs LL comparison.
