# Supplementary material for: Transcriptomic Profiling of Psoriatic Lesions by Tape-Stripping Reveals Site-Specific Differences
Source: J Clin Med. 2026 May 22;15(11):4034. doi: 10.3390/jcm15114034 (PMC13258650; doi:10.3390/jcm15114034)
Supplement: Supplementary file 1 [file jcm-15-04034-s001.zip › Supplementary Table S1.pdf]

Table S1: Characteristics of psoriatic patients

Characteristic	Psoriasis patients (N = 24)
**Age (mean ± SD)**	47.8 ± 15.7
**Sex**	
Female	9 (37.5%)
Male	15 (62.5%)
**BMI [1] (mean ± SD) [kg/m^2]**	30.2 ± 6.5
**BSA [2] (mean ± SD) [%]**	15.3 ± 12.5
**Age at diagnosis (mean ± SD)**	31.4 ± 16.2
**Comorbidities**	
Yes	11 (45.8%)
No	13 (54.2%)
**Family history of psoriasis**	
Yes	10 (41.7%)
No	13 (54.2%)
Missing	1 (4.2%)
**Smoking**	
Yes	4 (16.7%)
No	15 (62.5%)
Missing	5 (20.8%)
**Use of alcohol**	
Yes	1 (4.2%)
No	19 (79.2%)
Missing	4 (16.7%)

[1] Body Mass Index [2] Body Surface Area
